# SERS-based sensing of heavy metal ions and methylene blue using nanosecond laser ablated silver nanoparticles

**DOI:** 10.1039/d6ra05127h

**Published:** 2026-08-25

**Authors:** Pavithra K, S. Swaroop, Venugopal Rao Soma, Anusha P. T

**Affiliations:** a Department of Physics, School of Advanced Sciences, Vellore Institute of Technology (VIT) Vellore 632014 Tamil Nadu India sathyaswaroop.n.r@vit.ac.in; b Department of Physics, Kannur University, Swami Anandatheertha Campus-Payyanur Kannur 670327 India anushapt@kannuruniv.ac.in; c School of Physics and DIA-CoE (Formerly ACRHEM), University of Hyderabad Hyderabad 500046 India

## Abstract

Ligand-free silver nanoparticles (Ag NPs) were synthesized by nanosecond (ns) pulsed laser ablation of a bulk silver target in double distilled water (DW). The surface plasmon resonance (SPR) peak at 407 nm was observed from the UV-visible absorption spectrum. XRD and SAED analysis confirmed the crystallinity of face-centered cubic Ag NPs. The nanoparticle size with an average diameter of 21 ± 6 nm was confirmed from the TEM images. The obtained Ag NPs were employed as SERS substrates for the detection of dye molecule methylene blue (MB), and heavy metal contaminants such as lead nitrate and cadmium chloride. The SERS enhancement was dominated by electromagnetic mechanisms, with additional contributions from charge-transfer interactions and plasmon-mediated interfacial effects. The analytical enhancement factors were estimated to be ∼10^9^ for MB and ∼10^10^ for lead nitrate and cadmium chloride and detection limits down to picomolar (pM) concentrations were achieved without any additional chemical functionalization. These findings highlight the practical applicability of laser ablated Ag NPs as highly sensitive SERS substrates for detection of organic pollutants and toxic metal ions.

## Abstract 2

Highly sensitive SERS-based detection of heavy metal ions and methylene blue using silver nanoparticles synthesized *via* nanosecond laser ablation offering potential applications in trace-level analysis of environmentally hazardous contaminants.

## Graphical abstract

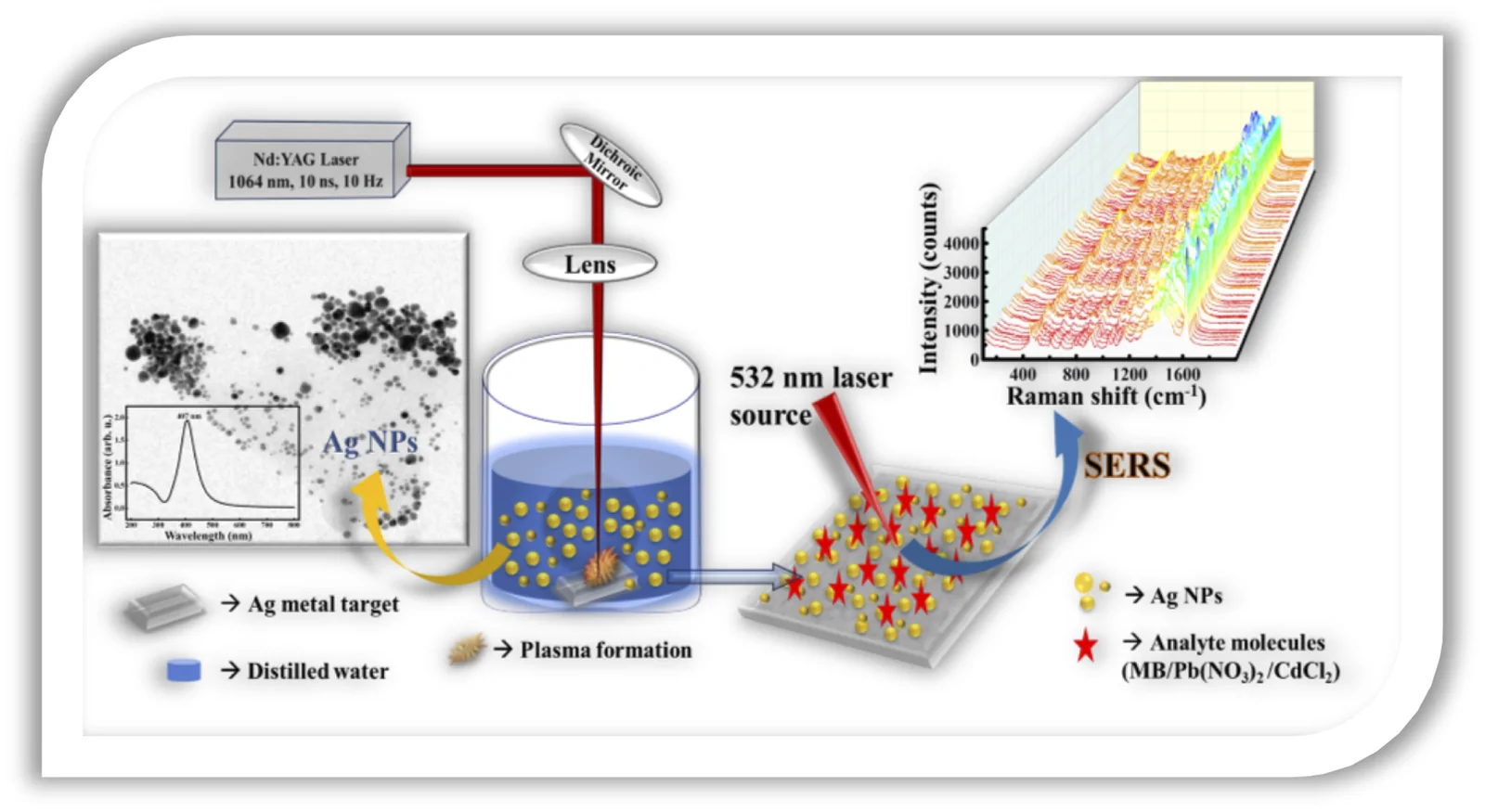

## 1 Introduction

Surface-enhanced Raman Spectroscopy (SERS) is an advanced optical sensing technique that enables ultrasensitive detection of trace-level analytes, owing to its widespread applications in environmental monitoring,^1,2^ biomedical diagnostics,^3,4^ food safety^5^ and chemical sensing.^6^ Since the observation of enhanced Raman signals on roughened Ag electrodes by Fleischmann *et al.* in 1974 ^7^ and subsequent theoretical foundations by Jeanmaire and Van Duyne,^8^ the SERS technique has relied on localized surface plasmon resonance (LSPR), which is the fundamental principle involved in enhancing Raman signals. SERS is a powerful spectroscopic technique that enables ultrasensitive molecular detection by amplifying weak Raman signals through localized electromagnetic fields generated near plasmonic nanostructures. The SERS enhancement mechanism is generally attributed to two primary contributions: (i) the electromagnetic (EM) enhancement, originating from intense near-fields localized at plasmonic “hot spots” and (ii) the chemical enhancement, associated with charge-transfer interactions between the adsorbed analyte and the metal surface.^9,10^ The efficiency and reproducibility of SERS enhancement depend on the properties of the plasmonic substrate, including the size of nanoparticles, morphology, crystallinity, surface purity of the substrate and its interaction with analyte molecules. The choice of plasmonic material and nanoparticle synthesis method plays an important role in determining the performance of the SERS substrate. Noble metal nanoparticles like Ag, Au and Cu have attracted significant attention due to their unique optical properties arising from LSPR, which results from the collective oscillation of conduction electrons under electromagnetic excitation.^11^ Among noble metals, silver nanoparticles (Ag NPs) are particularly interesting because of their strong plasmonic response in the visible region, high electromagnetic field enhancement, and comparatively low optical losses. These properties make Ag NPs highly suitable for applications in SERS, chemical sensing, photocatalysis, and environmental monitoring.^12^ However, conventional chemical synthesis methods often require stabilizing or capping agents, which block active surface sites and hinder analyte adsorption, thereby reducing SERS efficiency and reproducibility.^13,14^ The pulsed laser ablation in liquid (PLAL) technique has emerged as a clean, surfactant-free physical approach for producing colloidal metal nanoparticles with high purity, well-defined crystallinity and tunable size distribution. The absence of chemical contaminants makes PLAL-generated nanoparticles particularly attractive for SERS applications, where direct analyte-metal interactions are essential. Recently, it has been demonstrated that machine learning-optimized LAL enables environmentally sustainable synthesis of surface-clean Au nanoparticles, which exhibited enhanced surface-related properties and stronger plasmonic properties that are not achievable by wet chemical methods.^15^ In PLAL, the target is irradiated with high-energy laser pulses which induces the plasma formation at the surface of the target, followed by rapid cooling and nucleation, yielding high purity nanoparticles without any reducing agents or stabilizers.^16,17^ Different nanostructures are possible with chemical synthesis methods, however, the usage of stabilizers can reduce the plasmonic activity of the NPs. One of the unique features of Ag NPs which are produced by LAL is that they are spherical and ligand free. This makes the NPs interact with the analyte molecule more effectively as NPs get more ligand-free area for the hotspot generation. Baruah *et al.* have recently synthesized Ag NPs by pulsed laser ablation in distilled water and reported the potential of Ag NPs as SERS substrates to enhance the Raman signal of a bioactive furanoflavonoid, karanjin.^18^ Qayyum *et al.* have reported the synthesis of Ag NPs by ns PLAL for the detection of toxic dyes (*e.g.*, methylene blue and methyl orange) using the SERS technique.^19^ Ondieki *et al.*^20^ have systematically optimized the ablation parameters to enhance the suitability of Ag NPs as effective SERS substrates. They have demonstrated that the inherent plasmonic properties of Ag NPs contribute to enhancement in Raman signal, even in complex matrix such as rat's blood. Their findings also indicate that the Ag NPs can be effectively coupled with low-concentration target analytes for SERS. Bimetallic NPs are also widely used as SERS substrates for detection of dyes, explosives, pesticides.^21–23^ Kadavath *et al.* have used bimetallic Ag Au NPs for the detection of methylene blue (MB) and Rhodamine 6G.^22^ S. S. B. Moram *et al.* have reported the synthesis of Ag–Cu alloy NPs achieved by femtosecond laser ablation for the SERS sensing of dye molecule MB and explosives sensing of picric acid and ammonium nitrate.^23^ Methylene blue [C_16_H_18_ClN_3_S, MB], a dye molecule, is commonly employed as a model probe molecule in SERS studies due to its well-defined Raman fingerprint, strong adsorption on NPs surfaces, and relevance as an environmental pollutant.

Beyond molecular sensing, there is increasing interest toward the detection of inorganic ions and heavy metal contaminants, which are of major concern for environmental and public health. Heavy metal ions such as Pb^2+^ and Cd^2+^ and nitrate ions are highly toxic even at low concentrations and can accumulate in ecosystems, posing serious risks to human health and also to aquatic species.^24^ Heavy metal ions can be detected by conventional analytical techniques such as atomic absorption spectrometry (AAS),^25,26^ inductively coupled plasma mass spectrometry (ICP-MS),^27^ X-ray absorption spectrometry,^28^ and chromatography.^29^ SERS has emerged as non-invasive analytical technique for ultra-trace detection in food analysis and bioassay applications.^30,31^ However, detecting such ions using the SERS technique is challenging because many inorganic ions lack strong intrinsic Raman-active vibrational modes. Detection of heavy metal ions relies on plasmon-induced interfacial effects, including ion adsorption, modulation of the local dielectric environment, alterations in surface charge distribution at the NPs-analyte interface, rather than direct vibrational enhancement. These effects modify the localized electromagnetic field distribution and enable the enhancement of weak ionic Raman signatures, thereby extending the practical applicability of SERS beyond conventional molecular analytes. Nitrate ions originating from lead nitrate exhibit characteristic Raman modes that can be effectively enhanced by plasmonic substrates, enabling sensitive detection of nitrate species. Kadavath *et al.* reported the SERS detection of lead nitrate in water using Ag coated silicon substrates decorated with AgAu bimetallic nanoparticles synthesised by laser ablation in water as SERS substrates.^47^ However, incorporation of second metal may alter the plasmonic and dielectric properties of the substrate resulting change in the sensitivity.

Detection of heavy metals in existing literature mostly includes functionalized or immobilized nanoparticles and aptamer-based sensing.^32,49,50,52^ However, direct SERS-based detection of lead nitrate [(PbNO_3_)_2_] and cadmium chloride (CdCl_2_) salts without any functionalization remains largely unexplored. Moreover, Ag NPs which are synthesized by PLAL has not been investigated for sensing MB, lead nitrate and cadmium-based pollutants. Though multitudes of reports available for alloy-based NPs under PLAL, pure Ag NPs has been used in the current study to get maximum SERS sensitivity when compared with alloy-based substrates. This selection was initiated by the exceptional, intense LSPR peak of Ag NPs in the visible region. In this study, we report the synthesis of Ag NPs *via* nanosecond pulsed laser ablation in distilled water and their application as efficient SERS substrates for detection of methylene blue, lead nitrate, and cadmium chloride. This study demonstrates the feasibility of heavy-metal detection without surface functionalization and highlights the sensing capability at picomolar concentrations of analytes using ns laser ablated Ag NPs which serve as potential SERS substrates for environmental monitoring. The structural, optical, and elemental properties of the synthesized Ag NPs were systematically characterized using UV-visible spectroscopy, X-ray diffraction (XRD), transmission electron microscopy (TEM), selected area electron diffraction (SAED), field emission scanning electron microscopy with energy dispersive X-ray spectroscopy (FESEM-EDX) and Raman spectroscopy. This work focuses on direct detection of a dye molecule (MB) and heavy metal salts [(PbNO_3_)_2_ and CdCl_2_] using Ag NPs without any functionalization of nanoparticles and chemical surfactants. The SERS performance was evaluated in terms of analytical enhancement factor (AEF), limit of detection (LOD), limit of quantification (LOQ), relative standard deviation (RSD) and signal reproducibility. This study demonstrates the capability of laser-ablated Ag NPs without any additional functionalization to serve as multifunctional, contamination-free SERS platforms for environmental sensing.

## 2 Experimental details

### 2.1 Synthesis of the Ag NPs

**Fig. 1:**
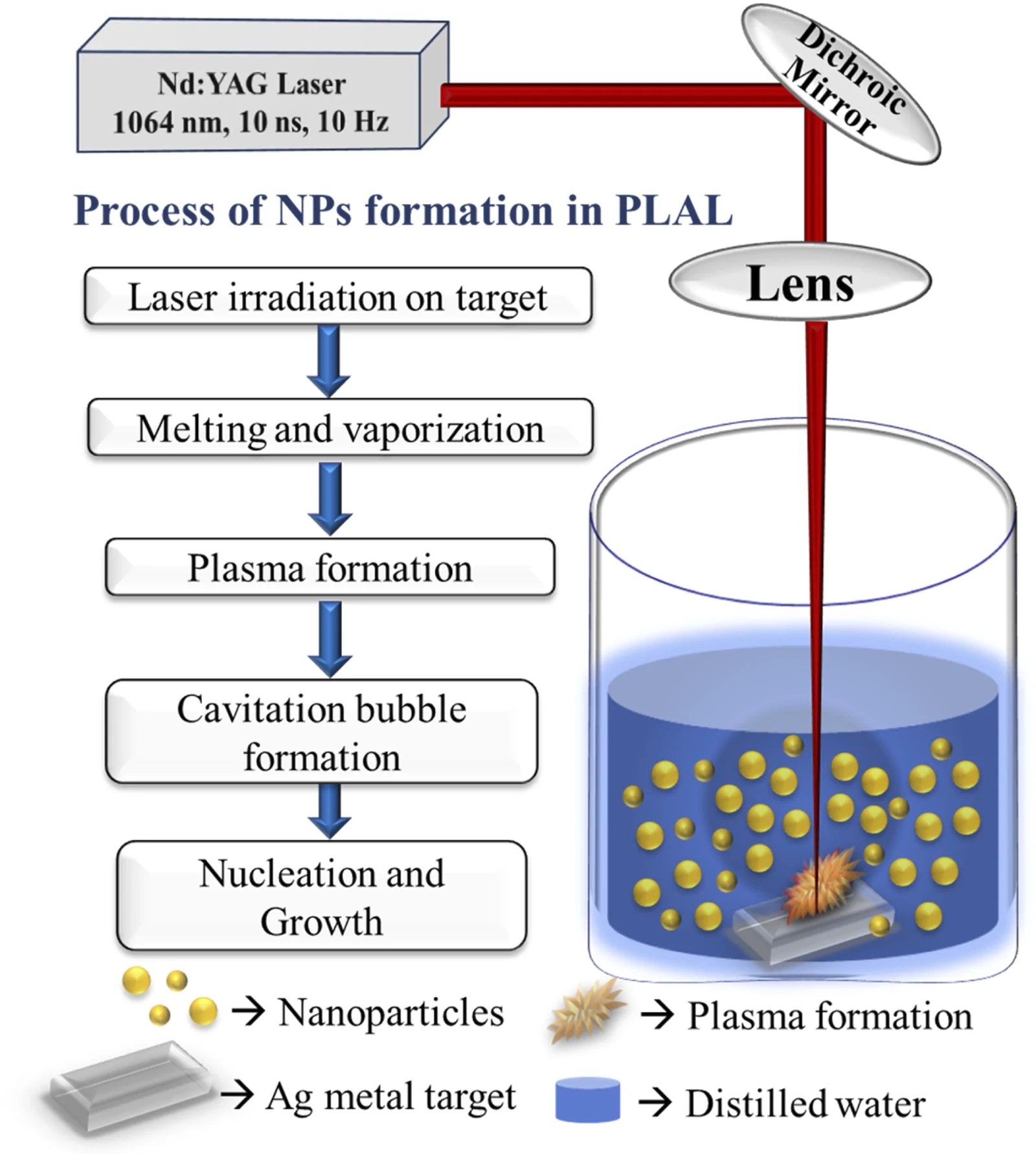
Experimental setup of pulsed laser ablation in liquid (PLAL) and process involved in formation of nanoparticles.

The experimental setup for synthesis of Ag NPs with the processes involved in ns PLAL is shown in Fig. 1. Ag metal plate (10 g 999 silver bar, Bangalore Refinery) with dimensions 1 mm thickness, 2.3 cm breadth, and 4 cm length was used as a target for ablation. Prior to laser irradiation, Ag metal plate was polished to remove the surface contaminants followed by cleaning with acetone and rinsed thoroughly with double distilled water (DW). The cleaned Ag plate was placed in the bottom of 250 mL glass beaker filled with 30 mL DW. The laser ablation was carried out using Q-switched Nd:YAG laser (Litron; Model No: LPY674-G10) operated at the fundamental wavelength of 1064 nm, with 10 Hz repetition rate and a pulse duration of 10 ns. The laser pulse energy was maintained at 265 mJ (1.35 J cm^−2^ fluence) for ablation with spot diameter of 5 mm. The Ag target was ablated for 1 hour and the sample was moved every 5 minutes to prevent continuous irradiation of laser pulse on the same spot. During laser-metal interaction, high temperature and high-pressure plasma plume was generated at the target-liquid interface. The liquid medium confines the expansion of plasma plume and its quenching in surrounding liquid medium, followed by the formation of cavitation bubble. After few micro seconds, the subsequent formation and collapse of cavitation bubbles lead to rapid cooling of ablated species, enabling nucleation and growth of Ag NPs in the liquid medium.^33,34^ The resulting Ag NPs colloidal solution was collected and used for further characterizations.

### 2.2 Instrumentation

The absorption spectra of synthesised Ag NPs were measured by JASCO V-670 UV-vis-NIR spectrophotometer. Crystal structure of Ag NPs was recorded using Bruker D8 Advance, Panalytical X'Pert^3^ (Cu Kα radiation, *λ* = 1.54 Å). 50 µL of Ag NPs solution was drop-casted and dried on cleaned glass substrate for XRD analysis. The size and shape of Ag NPs were confirmed from TEM operated at 200 kV (FEI-TECNAI, Model: G2-20 TWIN). Ag NPs solution was deposited on the carbon coated copper mesh grid and dried for TEM measurement. The size of nanoparticles was obtained using Image J software. Elemental mapping and EDX analysis of Ag NPs were done using FESEM (Thermo Fisher; Model:chemFEI QUANTA 250 FEG). The Raman measurements were performed using a 532 nm laser as an excitation source (Anton Paar, Cora 5001 DUAL) with ∼50 mW power. SERS mapping measurements were recorded using confocal Raman spectrometer (LABRAM HR EVO) with 532 nm laser source. For the SERS studies, the substrates were prepared by drop-casting 50 µL of laser ablated Ag NPs solution onto the cleaned glass substrates followed by drying on hotplate at ∼45 °C. The analyte molecules methylene blue (MB), lead nitrate (PbNO_3_)_2_ and cadmium chloride (CdCl_2_) were prepared as stock solutions at 1.5 mM, 0.02 M and 0.02 M concentration respectively. Analyte molecules with concentration ranging from 10^−3^ M to 10^−12^ M were prepared by serial dilution of respective stock solutions. 20 µL of prepared analyte solutions were then coated by drop cast method on the dried Ag NPs substrates for SERS measurements.

## 3 Results and discussion

### 3.1 UV-visible absorption spectrum of Ag NPs

**Fig. 2:**
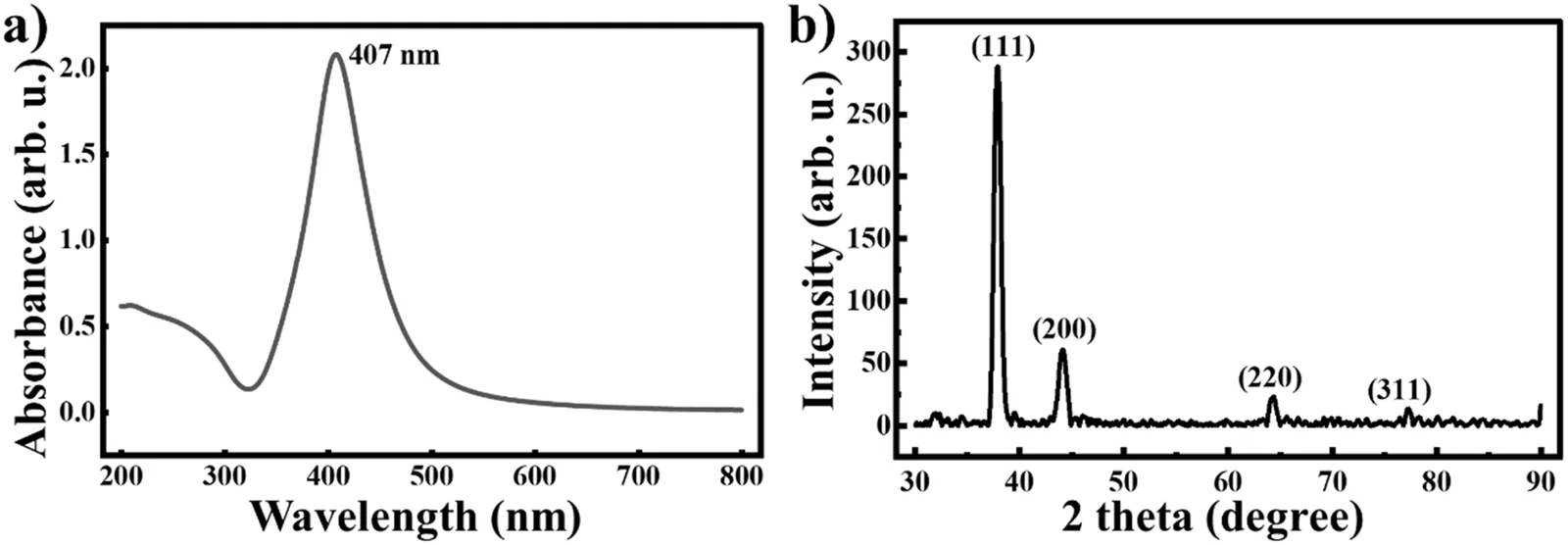
(a) UV-visible absorption spectrum (b) XRD spectrum of Ag NPs synthesised by PLAL.

**Fig. 3:**
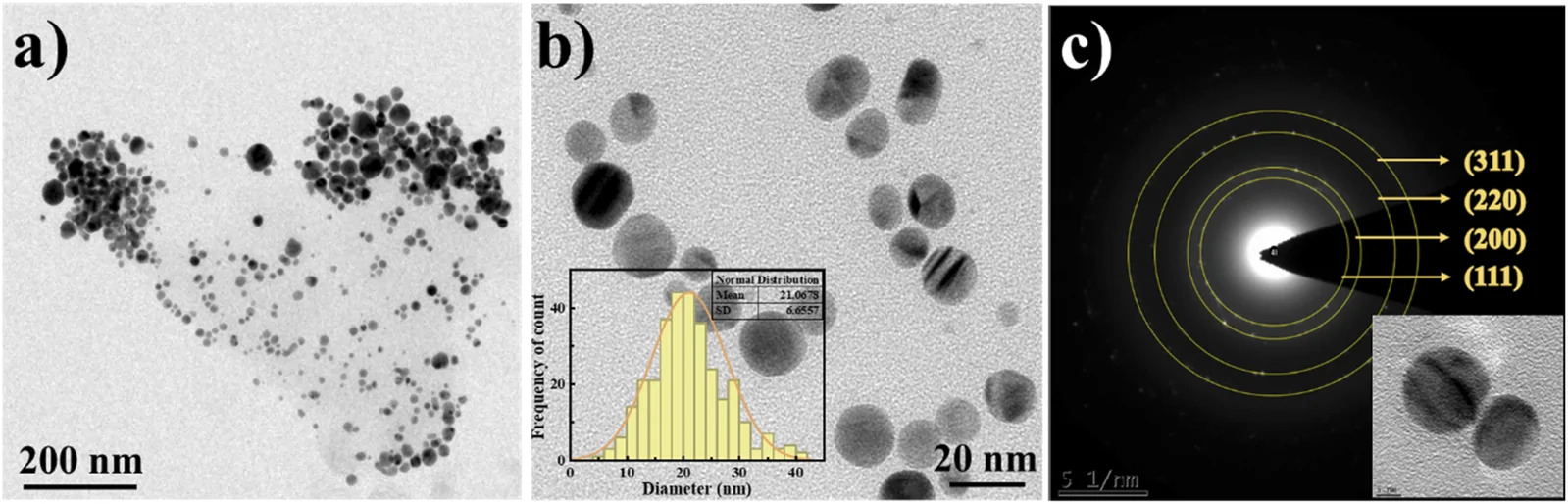
(a) TEM image of Ag NPs, (b) TEM image of Ag NPs with size distribution depicted in the inset and (c) SAED pattern with HRTEM image of the Ag NPs in the inset.

Fig. 2(a) represents the UV-visible absorption spectrum of Ag NPs synthesised by ns laser ablation in DW. The maximum absorbance of Ag NPs is centered at 407 nm, which corresponds to surface plasmon resonance (SPR) of metallic Ag NPs. The SPR peak (407 nm) confirms the formation of Ag NPs in the liquid medium. The SPR band arises from the collective oscillation of conduction band electrons at the surface of nanoparticles under electromagnetic excitation. The SPR peak at 407 nm suggests the formation of small and spherical Ag nanoparticles with narrow size distribution. TEM analysis also reveals the size distribution of Ag NPs [data presented in Fig. 3(b) in the subsequent section]. Various literature reports have shown that the Ag NPs typically exhibit SPR peak around 400–420 nm for small, spherical Ag NPs using ns pulsed lasers.^19,33,35,36^ Qayyum *et al.*^19^ reported the SPR peak at 405 nm for Ag NPs synthesised by ns laser ablation in double distilled water attributing to spherical Ag NPs. Amendola and Meneghetti^33^ observed the SPR peaks at 400 nm for laser-generated Ag NPs and emphasized that the absence of external capping agents results in pure nanoparticles and sharp plasmonic features. The SPR peak at 407 nm obtained in the present study closely matches these findings, indicating comparable nanoparticle size and morphology. Tanoira *et al.*^36^ reported SPR peak at 400 nm of spherical Ag NPs synthesised by nanosecond and picosecond laser ablation. The variation of SPR peak in 408 nm to 412 nm spectral range with respect to ablation time was reported by Juma *et al.*^37^ Laser based synthesis produces high purity and ligand-free NPs, resulting in sharper and symmetric SPR peak, as observed in this work. Fig. S1 of the SI shows the absorption spectrum of 1.5 mM MB dispersed in distilled water. Absorption spectra of Pb(NO_3_)_2_ and CdCl_2_ dispersed in distilled water at 0.02 M concentration is shown in Fig. S2 of the SI.

### 3.2 XRD analysis of Ag NPs

**Fig. 4:**
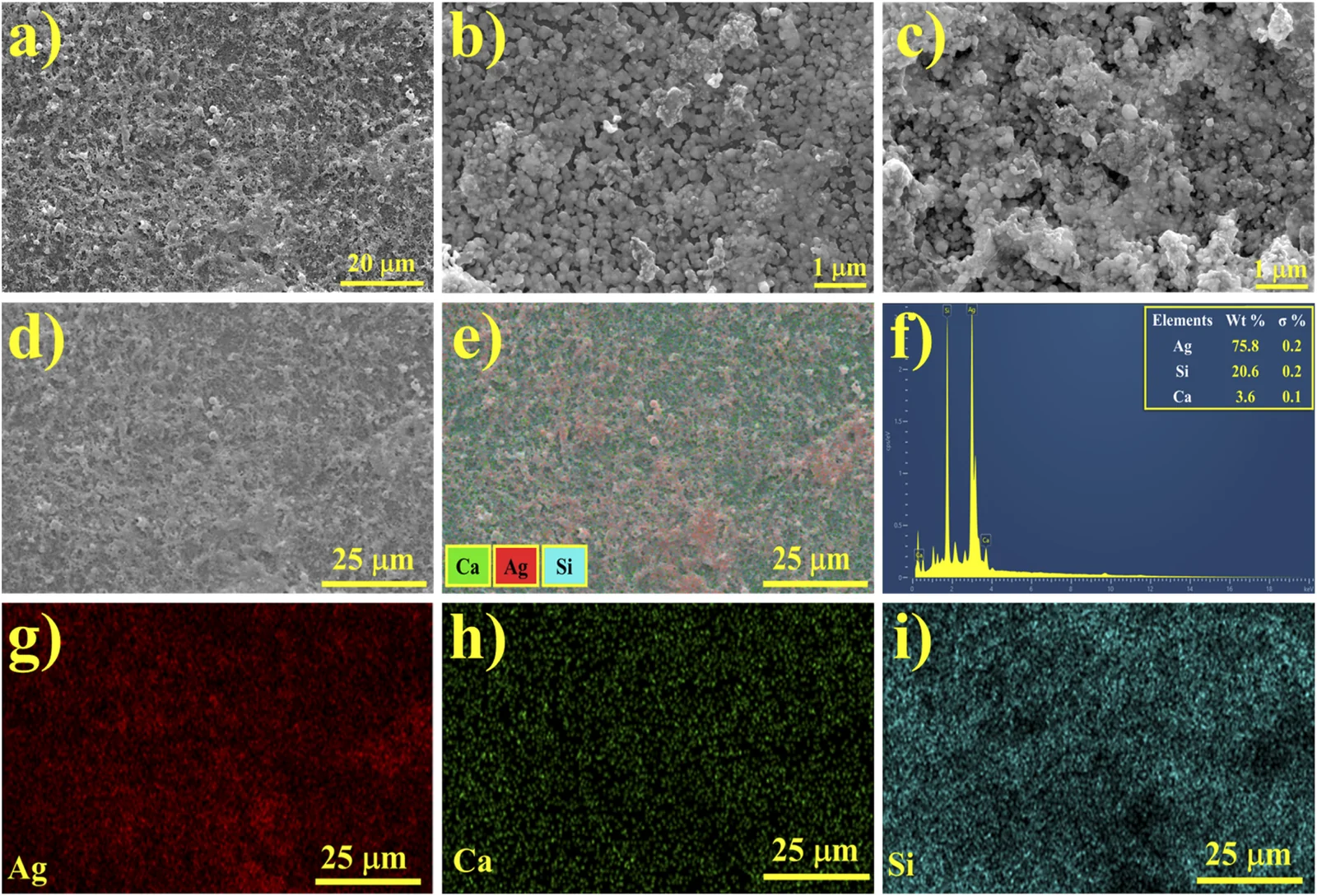
(a)–(c) FESEM images, (d) and (e) FESEM – EDS mapping, (f) EDX spectrum, (g)–(i) elemental mapping of Ag NPs coated on a glass substrate.

Fig. 2(b) shows the XRD pattern of Ag NPs synthesised by ns PLAL which is obtained by drop casting 50 µL of Ag NPs solution on glass substrate. The diffraction pattern exhibits well – defined peaks attributing to the planes (111), (200), (220) and (311) at 37.8°, 44.12°, 64.35° and 77.35° respectively [JCPDS file number: 04-0783] of face-centered cubic (fcc) Ag.^38,39^ The corresponding *d*-spacing values were calculated to be 2.3 Å, 2.0 Å, 1.4 Å and 1.2 Å, respectively. The absence of additional peaks confirms that no oxide or secondary phases were formed during the ablation process, which is also confirmed by the SAED analysis from HRTEM data [Fig. 3(c)] and elemental mapping from FESEM data (Fig. 4).

### 3.3 TEM of Ag NPs

The size and shape of laser ablated Ag NPs were confirmed using TEM analysis. Fig. 3(a) and (b) depict the morphology of Ag NPs with their size distribution histogram shown in the inset. High resolution transmission electron microscope (HRTEM) image with selected area electron diffraction (SAED) pattern is depicted in Fig. 3(c). The average diameter of the Ag NPs was quantitatively evaluated by measuring the size of ∼300 nanoparticles using image J software. TEM images revealed that the Ag NPs are spherical and are well dispersed. Histogram analysis of TEM shows that the average particle diameter is ∼21 nm with a standard deviation of ∼6 nm, confirming narrow size distribution of Ag NPs. The particle size obtained from TEM correlates with SPR peak observed at 407 nm in the absorption spectrum. HRTEM image reveals the distinct lattice fringes within individual Ag NPs with measured lattice spacing of 2.3 Å which is consistent with (111) plane of fcc structure of Ag. Concentric diffraction rings in SAED pattern corresponds to (111), (200), (220) and (311) planes of fcc Ag with 2.3 Å, 2.0 Å, 1.4 Å and 1.2 Å *d*-spacing values respectively.^23,39^ The *d*-spacing values calculated from SAED pattern goes well in agreement with the *d*-spacing calculated from XRD analysis. TEM, HRTEM and SAED analysis confirms that the obtained Ag NPs are spherical with narrow size distribution, uniform and crystalline. This also confirms the consistency between the optical and morphological characterization of Ag NPs by correlating the average particle size of ∼21 nm to the observed SPR peak at 407 nm.

### 3.4 FESEM analysis

Fig. 4(a)–(c) illustrate the FESEM images of Ag NPs. The elemental mapping of synthesised Ag NPs is shown in Fig. 4(d) and (e). EDX spectrum [Fig. 4(f)] shows the presence of Ag NPs by exhibiting a prominent Ag Lα peak at ∼3 keV. The elemental mapping is represented in Fig. 4(g)–(i) which shows the presence of Ag NPs throughout the scanned region. Since Ag NPs are coated on the glass substrate for the measurements, presence of calcium and silicon is observed from the elemental analysis. The combined results of TEM, HRTEM, SAED, XRD and UV-vis confirms that the ns PLAL is an effective method to produce crystalline Ag NPs with controlled size and strong plasmonic behaviour.

Though Ag NPs formed by PLAL are spherical in shape, high AEF depends on the collective effect of plasmonic hotspots, morphology, relatively clean surface of the nanoparticles and effective adsorption of the analyte molecules onto the surface of Ag NPs. The average size of 21 nm with SD of 6.6 nm is obtained by TEM analysis (Fig. 3) of nanoparticles as-synthesised colloid and reflect size distribution of the Ag NPs. However, the FESEM analysis (Fig. 4) represents the Ag NPs coated on the glass substrate which is used for SERS measurements, showing the close arrangement of NPs. This clustered arrangement of NPs facilitates strong plasmon coupling and formation of multiple interparticle hotspots, which contributes to high AEF. Unlike other nanostructures (such as nano stars, nanorods, nanowires, nano flowers) which generates intense electromagnetic fields primarily at sharp tips and edges which are synthesised by chemical methods involving surfactants and stabilizing agents, spherical Ag NPs synthesised by laser ablation in water exhibit strong enhancement and high AEF when they are closely assembled into clusters, where their surfactant-free and relatively clean surface facilitates effective analyte interaction and hotspot formation.

### 3.5 SERS activity of Ag NPs

The plasmonic efficiency of laser ablated Ag NPs were evaluated by SERS measurements. SERS performance of Ag NPs as substrate was investigated by recording the Raman spectrum of the analyte molecules MB (1.5 mM), Pb(NO_3_)_2_ (0.02 M), CdCl_2_ (0.02 M) using 532 nm laser source. Firstly, the stock solutions were prepared at a higher concentration. Then the desired concentrations of 1 mM, 1 µM, 15 nM, 1 nM, 20 pM, 15 pM, 10 pM, 5 pM, 3 pM and 1 pM solution were prepared by serial dilution method. 50 µL of Ag NPs solution was first coated on the pre-cleaned glass substrate (1 × 1 cm) and allowed to dry on hot plate. Then 20 µL of analyte solution was drop casted on the Ag NPs substrate followed by drying. Raman spectrum of the analyte molecules drop casted on the glass substrate were recorded at higher concentration and this is considered as reference Raman spectrum. SERS spectra of low concentration analyte molecules were then recorded. The average of five measurements taken at different spots is plotted and presented here for SERS analysis. SERS performance for each analyte is estimated by AEF for the most prominent Raman bands using the formula:1
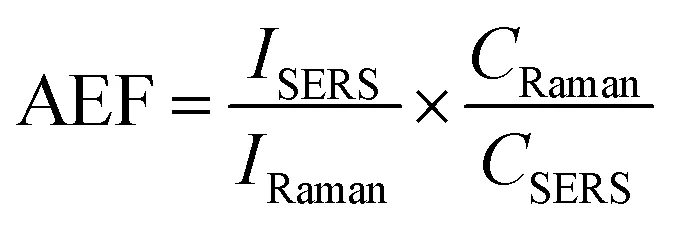
where *I*_SERS_ is the SERS intensity of analyte with Ag NPs; *I*_Raman_ is the Raman intensity of analyte molecule without Ag NPs; *C*_Raman_ is the concentration of the analyte molecule used to record normal Raman spectrum; *C*_SERS_ is the concentration of the analyte molecule used for SERS analysis with Ag NPs.^40,43^

Analytical performance of the SERS substrates was checked by performing quantitative analysis to evaluate the sensitivity (LOD), LOQ and RSD. The sensitivity of Ag NPs SERS substrate was found by LOD value which is calculated using the formula 
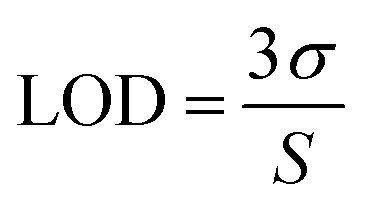
, LOQ is given as 
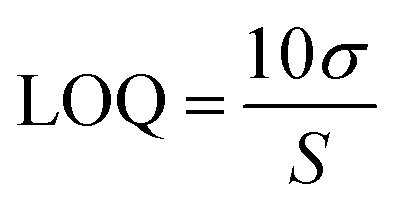
 where *σ* is the standard deviation of intensity values from bare substrate and *S* is the slope value from calibration graph.^41^ Uniformity of the substrate is a prominent factor in determining the practical applications of SERS. To evaluate the uniformity of SERS substrates, SERS mapping analysis was performed at 1 pM concentration of the analytes. Fig. S6 of the SI presents the SERS intensity mapping images of methylene blue, Pb(NO_3_)_2_ and CdCl_2_ at 1 pM concentration with Ag NPs, which confirms the homogeneity of the substrates even at 1 pM concentration. The figures show almost same signal quality which emphasis that the substrate is uniform. RSD values determine the signal homogeneity of SERS substrates which is calculated by using the formula 
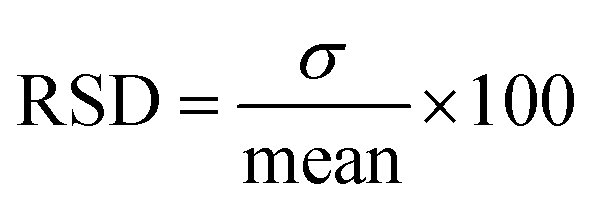
 where *σ* is the standard deviation of Raman intensity values and mean is the average of intensity values from mapping.

#### 3.5.1 Methylene blue SERS data

**Fig. 5:**
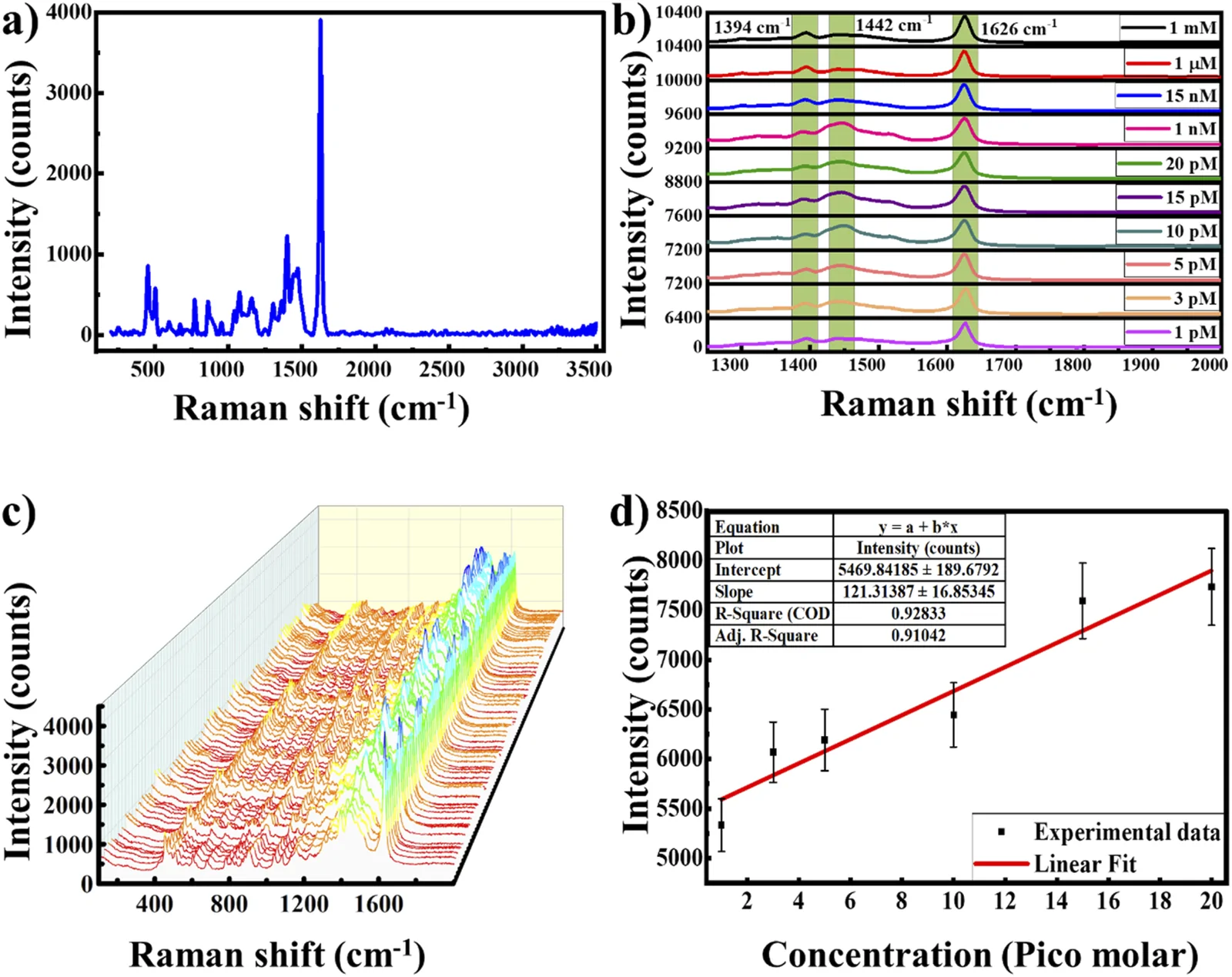
(a) Raman spectrum of MB at 1.5 mM concentration (b) SERS spectra of MB in different concentrations (1 mM to 1 pM) with Ag NPs (maximum scale value is mentioned in *y*-axis), (c) SERS mapping data of MB (1 pM) at 86 different spots, (d) calibration graph of MB at 1626 cm^−1^ (Black dots represents experimental data with error bar and red line is fitted data).

MB was used as a probe molecule due to its strong and distinct Raman characteristic peaks. Fig. 5(a) shows the Raman spectrum of MB at 1.5 mM concentration recorded without Ag NPs. Raman bands at 1626 cm^−1^, 1394 cm^−1^, 1302 cm^−1^, 1442 cm^−1^, 1157 cm^−1^ and 450 cm^−1^ corresponding to C–C ring stretching, symmetric stretching of C–N bonds, in-plane ring deformation (C–H ring deformation), asymmetric stretching of C–N, C–H in-plane bending and skeletal deformation (C–N–C mode) respectively are observed. These band assignments are consistent with existing SERS studies on MB.^38,42,43^Fig. 5(b) shows the SERS spectra of MB with Ag NPs are taken for various concentrations of MB ranging from 1 mM to 1 pM. C–C ring stretching mode of MB at 1626 cm^−1^, C–N symmetrical stretch at 1394 cm^−1^ and C–N asymmetric stretching at 1442 cm^−1^ were the three major modes identified even at low concentration (1 pM). AEFs were calculated for these modes for different concentrations of MB and are presented in Table S1 with their intensity values. Enhancement factor was obtained in the order of 10^9^ for pM concentration of MB. Fig. 5(c) depicts the SERS signal homogeneity of MB collected from mapping data over 86 points on the substrate at 1 pM concentration which implies the reproducibility, reliability and uniformity of the SERS substrate. Figure S3 of the SI presents the SERS signal homogeneity graphs of MB at 20 pM, 15 pM, 10 pM, 5 pM and 3 pM concentrations taken at five random spots. The quantitative detection capability of SERS substrate was evaluated using the calibration graph [Fig. 5(d)] with intensity on *y*-axis and concentration of MB from 1 pM to 20 pM on *x*-axis for the most prominent characteristic band (1626 cm^−1^). The *R*^2^ value was found to be 0.92 which validates the detection capability of Ag NPs as SERS substrates. LOD was calculated to be 7.21 × 10^−12^ M, LOQ was found to be 24.05 × 10^−12^ M at picomolar concentrations of MB at 1626 cm^−1^. The RSD value was calculated to be 12% from SERS mapping. LOD, LOQ and RSD values indicates that, the laser-ablated Ag NPs as SERS substrates have excellent sensitivity for trace level detection of MB. Kadavath *et al.* have reported the EF of the order of 10^8^ and detection limit of 10^−12^ for MB with AgAu bimetallic nanoparticles.^39^ Vu *et al.* have achieved an EF of ∼1.6 × 10^6^ with MB concentration of 10^−7^ M and detection limit of 10^−7^ M^44^ with silver nanodecahedra prepared by photochemical route.

To further explore the applicability of laser-ablated Ag NPs substrates for environmental sensing, SERS measurements were performed using the heavy metal salt solutions of lead nitrate [Pb(NO_3_)_2_] and cadmium chloride (CdCl_2_) as analytes. Detection of these metal ions such as Pb^2+^, Cd^2+^ and also nitrate ion (NO_3_^−^) has become a research hotspot because of their toxicity, their presence in aqueous environments and their inability to degrade themselves which affects public health and ecological balance of the environment.^24,49^

#### 3.5.2 Lead nitrate SERS data

**Fig. 6:**
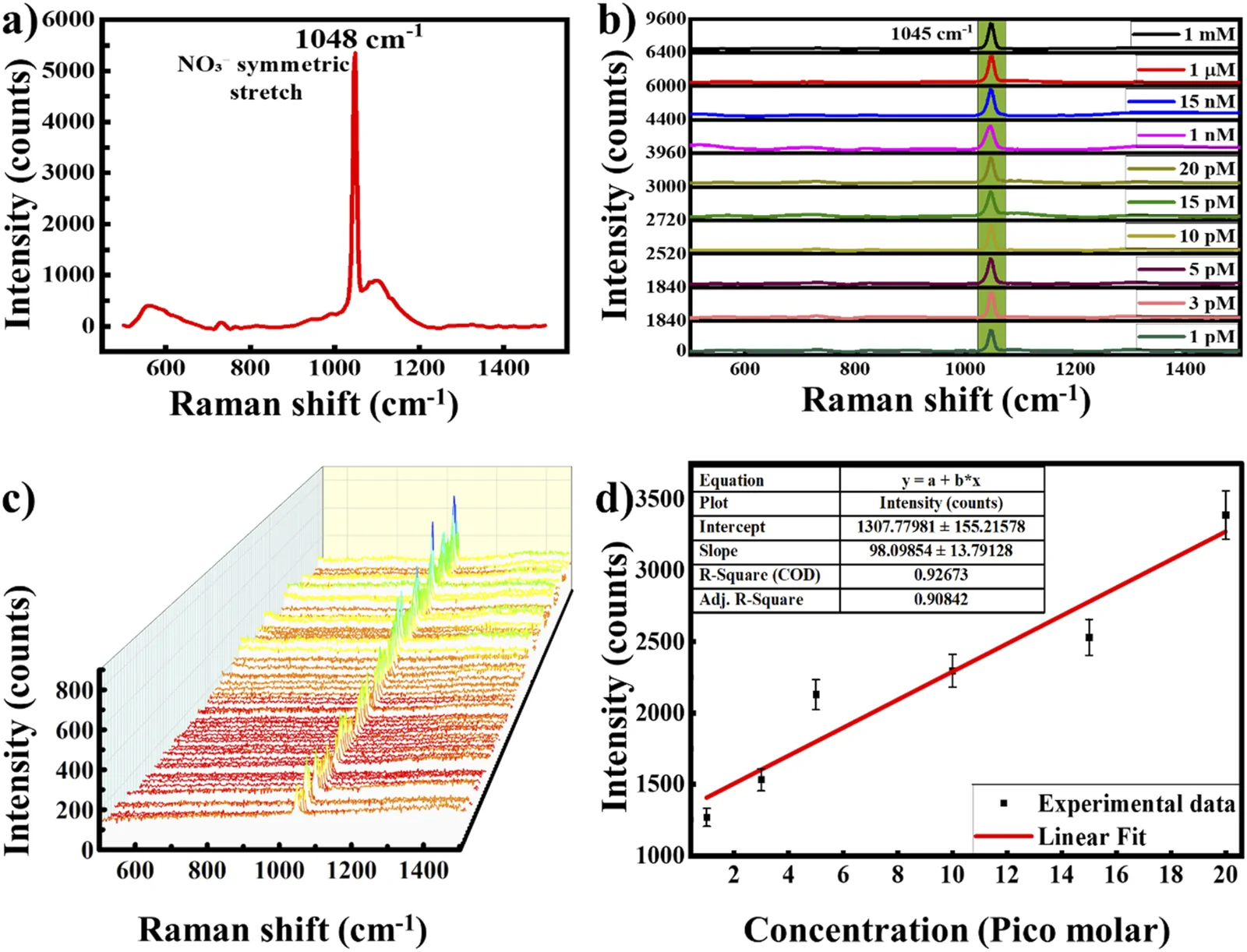
(a) The Raman spectrum of lead nitrate at 0.02 M concentration (b) SERS spectra of Pb(NO_3_)_2_ in different concentrations (1 mM to 1 pM) with Ag NPs (maximum scale value is mentioned in *y*-axis) (c) SERS mapping of 1 pM Pb(NO_3_)_2_ at 100 different points, (d) calibration graph of Pb(NO_3_)_2_ at 1045 cm^−1^ (Black dots represents experimental data with error bar and red line is fitted data).

Fig. 6(a) shows the Raman spectrum of lead nitrate at 0.02 M concentration. Prominent Raman band appeared at 1048 cm^−1^ corresponding to the symmetric stretch of nitrate ion.^45,46^ This band is significantly enhanced and a slight shift to 1045 cm^−1^ was observed when adsorbed onto Ag NPs as shown in Fig. 6(b), confirming the effective sensing ability of the SERS substrate towards nitrate ion detection. The strong SERS response of Pb(NO_3_)_2_ is dominated by the nitrate (NO_3_^−^) ion, which acts as the primary Raman-active species. SERS spectra of lead nitrate were recorded for different concentrations ranging from 1 mM to 1 pM. Enhancement factor was calculated for the prominent peak 1045 cm^−1^ and are listed in Table S2 for various concentrations with the SERS intensity values. AEF was estimated to be in the order of 10^10^ for pM concentration of lead nitrate. Fig. 6(c), shows the SERS mapping spectra of lead nitrate at 1 pM concentration taken at 100 different spots which confirms the repeatability and uniformity of the SERS substrate. Fig. S4 of the SI presents the SERS signal homogeneity graphs of lead nitrate at 20 pM, 15 pM, 10 pM, 5 pM and 3 pM concentrations taken at five random spots. Fig. 6(d) depicts the calibration graph of lead nitrate in pM concentration ranging from 1 pM to 20 pM for the characteristic nitrate ion stretching band at 1045 cm^−1^. The *R*^2^ value was found to be 0.92. The LOD, LOQ and RSD values were calculated to be 2.2 × 10^−12^ M, 7.3 × 10^−12^ M and 30% respectively. Upon adsorption onto the Ag nanoparticle surface, nitrate ions experience a significantly enhanced local electromagnetic field generated by localized LSPR. This results in a pronounced enhancement of the symmetric stretching mode near ∼1045 cm^−1^. The values are comparable with that of reports by J. K. Kadavath *et al.* in the SERS detection of lead nitrate in water with LOD of 1 pM using Ag coated silicon substrates decorated with AgAu bimetallic nanoparticles synthesised by laser ablation in water as SERS substrates.^47^

#### 3.5.3 Cadmium chloride SERS data

**Fig. 7:**
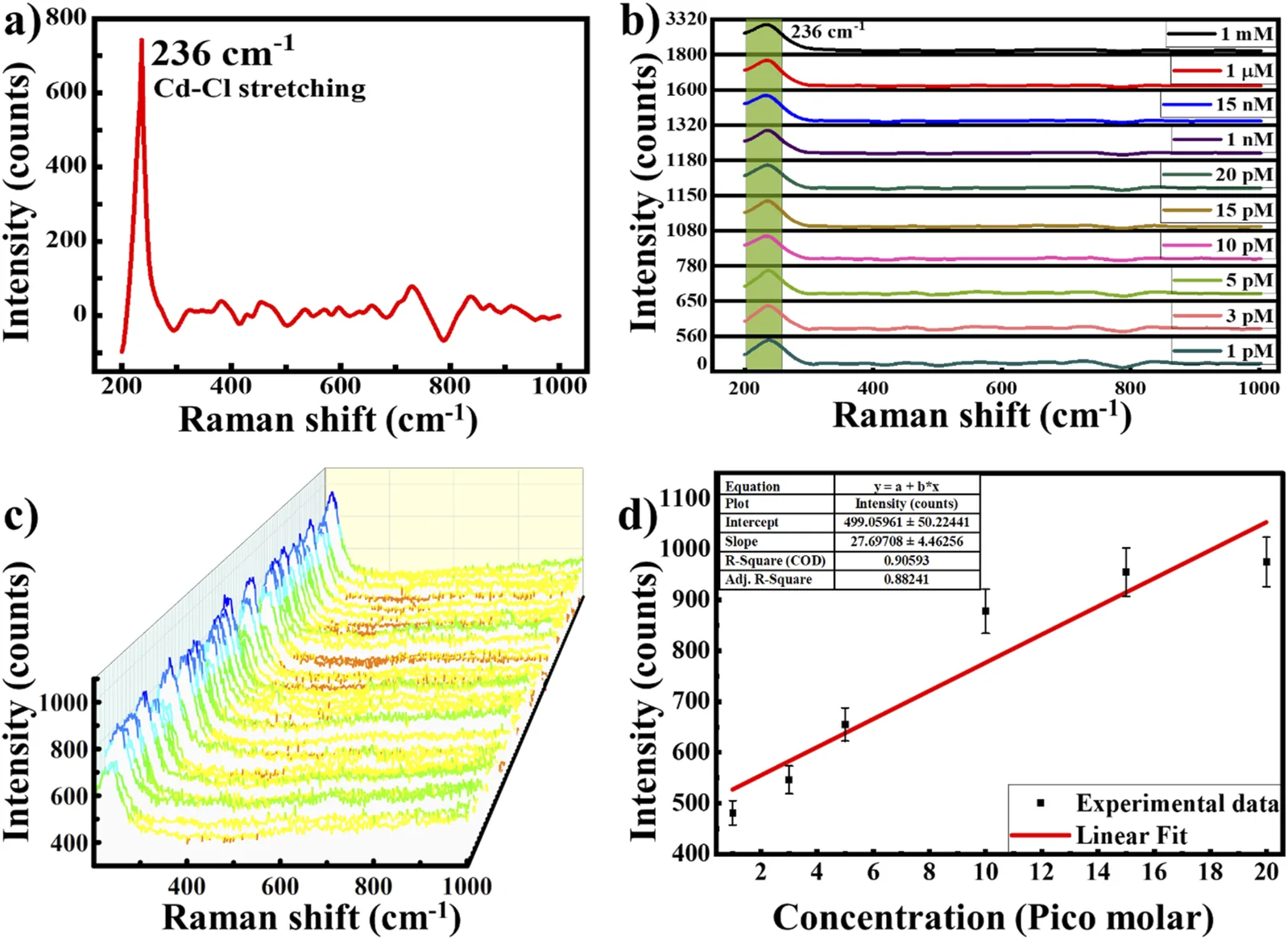
(a) Raman spectrum of CdCl_2_ (0.02 M concentration) (b) SERS spectra of CdCl_2_ in different concentrations (1 mM to 1 pM) with Ag NPs (maximum scale value is mentioned in *y*-axis) (c) SERS mapping of 1 pM CdCl_2_ at 77 different points (d) calibration graph of CdCl_2_ at 236 cm^−1^ (Black dots represents experimental data with error bar and red line is fitted data).

Fig. 7(a) shows the Raman spectrum of cadmium chloride at 0.02 M concentration. Prominent Raman band appeared at 236 cm^−1^ corresponding to the Cd–Cl stretching. This band is significantly enhanced when adsorbed onto Ag NPs, confirming the effective sensing ability of the substrate towards nitrate ion detection. The SERS spectra of CdCl_2_ are shown in Fig. 7(b). SERS spectra of cadmium chloride were recorded for different concentrations ranging from 1 mM to 1 pM. Enhancement factor was calculated for the prominent peak 236 cm^−1^ and are listed in Table S3 for various concentrations. AEF was found to be in the order of 10^10^ for pM concentration of CdCl_2_. Fig. 7(c) shows the SERS mapping spectra of CdCl_2_ at 1 pM concentration taken at 77 different spots which confirms the repeatability and uniformity of the SERS. Fig. S5 of the SI presents the SERS signal homogeneity graphs of cadmium chloride at 20 pM, 15 pM, 10 pM, 5 pM and 3 pM concentrations taken at five random spots. Fig. 7(d) depicts the calibration graph of CdCl_2_ in pM concentration from 1 pM to 20 pM. The *R*^2^ value was found to be 0.9. LOD, LOQ and RSD values were calculated to be 12.1 × 10^−12^ M, 40.5 × 10^−12^ M and 14% respectively. For cadmium chloride, the SERS enhancement was observed due to dominant electromagnetic enhancement mechanism. The enhancement in the SERS signal is attributed to the adsorption of Cd^2+^ ions and associated chloride ions onto the Ag NPs surface, which modifies the local electromagnetic field and facilitates Raman enhancement.

### 3.6 Stability studies

The stability of ns laser ablated Ag NPs as effective SERS substrates for sensing was studied using both UV-vis absorption and SERS spectroscopy. Fig. S7 (SI) shows the absorption spectra of Ag NPs recorded over the time interval of two months. The SPR peak of synthesised Ag NPs at 407 nm is shifted to 406 nm with a slight decrease in absorbance intensity after two months of storage. This negligible blue shift to 406 nm and a minor decrease in absorbance is due to the settling of nanoparticles over time, reducing the effective concentration of nanoparticles. The absence of significant red shift and broadening of SPR band indicates that there are no considerable aggregation and change in optical properties occurred during the storage period. Fig. S8 of the SI depicts the SERS spectra MB, Pb(NO_3_)_2_ and CdCl_2_ at a concentration of 1 pM as prepared and after 2 months of storage using Ag NPs substrates. The changes in SERS intensities and the peak shift of the SERS substrates were observed after storage of two months. Table S4 shows the AEF values at concentration of 1 pM of the SERS substrates as prepared and after storage of two months. After two months the SERS signal response has decreased by 10–40% only with appropriate storing mechanism. Observed changes are due to the reduction in effective nanoparticles concentration over storage, alteration in analyte adsorption and the interaction between analyte and Ag NPs over time. Though there are minor changes observed in optical and SERS characteristics during the storage of two months, the negligible SPR changes and considerable changes in SERS intensities indicates that the laser ablated Ag NPs remained relatively stable to serve as effective SERS substrates even at low concentration (1 pM).

## 4 Overview of mechanism involved in SERS

**Fig. 8:**
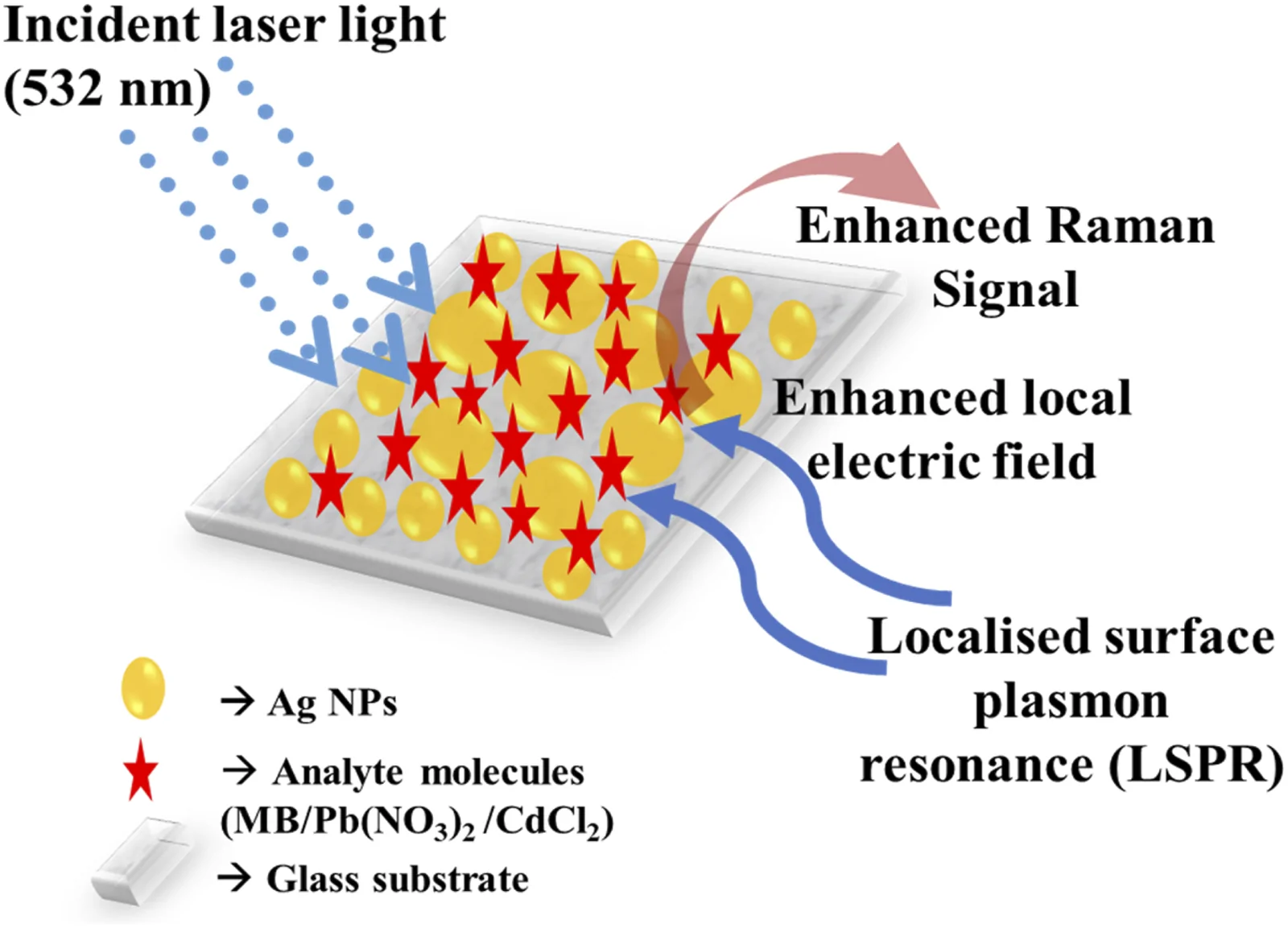
Schematic of the electromagnetic enhancement mechanism in SERS.

The SERS results obtained for MB, lead nitrate, and cadmium chloride collectively demonstrate the strong plasmonic activity and broad sensing capability of ns laser-ablated Ag NPs substrate. Despite the chemical and structural differences and the variation in Raman – active sites in the organic dye (MB) and inorganic ionic analytes (lead nitrate and cadmium chloride), significant Raman enhancement is observed for all three samples, indicating that electromagnetic enhancement governed by plasmonic effects is the dominant mechanism.^10,48^Fig. 8 shows the schematic of electromagnetic enhancement mechanism in SERS. This is the primary mechanism responsible for SERS enhancement, which arises from the excitation of LSPR in the Ag nanoparticles. When the Ag NPs are illuminated with the excitation laser (532 nm), collective oscillations of conduction electrons are induced, leading to the formation of strongly localized electromagnetic fields near the nanoparticle surface (hotspots).^9,48^ These enhanced fields amplify both the incident and scattered electromagnetic waves, resulting in Raman intensity enhancement proportional to |E|^4^.

For MB, the observed high EF (∼10^9^) and low LOD are attributed to a combination of electromagnetic enhancement and chemical (charge-transfer) enhancement. MB molecules adsorb efficiently onto the Ag NP surface through π-electron interactions and electrostatic attraction, leading to enhanced electromagnetic field. In addition to EM enhancement, charge-transfer interactions between the Ag Fermi level and the molecular orbitals of MB can modify the molecular polarizability, leading to further enhancement of specific vibrational modes.^42^

In the case of lead nitrate, Pb^2+^ ions interact weakly with Ag surface and, therefore, the SERS signal is dominated by the nitrate (NO_3_^−^) ion vibrational modes, particularly the symmetric stretching mode at 1045 cm^−1^. Unlike MB, nitrate ions do not possess extensive π-electron systems; therefore, their enhancement is primarily governed by the electromagnetic mechanism. Nitrate ions which are adsorbed near the Ag NP surface experience strong local field enhancement, which amplifies their Raman response despite their relatively weak intrinsic Raman cross-section. The clear observation of nitrate vibrational modes at picomolar concentrations indicates that the electromagnetic field generated by the Ag NPs is sufficiently strong to enhance even small inorganic ions.^45,46^

In cadmium chloride, the enhancement arises from the intrinsic vibrational characteristics of Cd^2+^ and Cl^−^ ions, which do not possess strong Raman-active internal vibrational modes. Unlike molecular analytes, these ions lack polarizable bonds that can efficiently couple to the incident electromagnetic field, limiting the contribution from direct molecular Raman scattering.^22^ However, detectable SERS signals are observed, indicating that the enhancement mechanism for CdCl_2_ is governed by indirect plasmon-ion interactions rather than intrinsic vibrational enhancement. One key contribution arises from the specific adsorption of Cd^2+^ ions onto the Ag nanoparticle surface is facilitated by electrostatic interactions between the positively charged metal ions and the negatively polarized regions of the Ag surface under plasmon excitation.^24^ This adsorption modifies the local dielectric environment at the metal-analyte interface, leading to changes in the plasmon resonance condition and localized field distribution. Despite the absence of strong intrinsic Raman modes, the ability to detect CdCl_2_ at pM concentrations without any functionalization highlights the exceptional sensitivity of the laser-ablated Ag NPs substrate. This sensitivity arises from the dominant electromagnetic enhancement, which is sufficiently strong enough to amplify weak Raman signal. A recent work by Liu *et al.* developed highly sensitive SERS-based microfluidic sensor for detection of Pb^2+^ and Cd^2+^ in seawater using volcanic crater-shaped array substrate fabricated by ultraviolet lithography and fs laser-induced gold nanoparticles doping in poly (methyl methacrylate). Using DNAzyme and aptamer-based recognition strategies, they have reported low detection limits of 10^−12^ M for Pb^2+^ and 10^−11^ M for Cd^2+^.^49^ S. S. R. Dasary *et al.* reported the detection of cadmium with enhancement factor ∼3.2 × 10^7^ in water samples using a Raman active Alizarin dye functionalized with gold nanoparticles.^50^

Table 1 presents the comparison of enhancement factors and LOD values for the selected analyte molecules with those reported in the existing literature. The presented outcomes reveals that the ligand-free Ag NPs synthesised by ns PLAL ensures direct adsorption of analyte molecules. Ag NPs substrate exhibited higher AEFs and low detection limits along with good uniformity and repeatability, revealing the homogeneity of SERS substrates even at low concentration (1 pM) due to smaller particle size, sharp SPR peak and also clean surfaces of Ag NPs SERS substrate (without surfactants and functionalization). This demonstrates the potential ability of laser ablated Ag NPs to serve as sensing platforms in SERS for the detection of toxic dye molecules and heavy metal contaminations for environmental monitoring.

**Table 1:** Comparison of EFs and LOD of MB, nitrate ion and cadmium

Substrate	Analyte (concentration)	Raman bands (cm^−1^)	EF	LOD	Ref.
Ag nanodecahedra	MB (10^−8^ M to 10^−4^ M)	1625	1.602 × 10^6^	10^−7^ M	44
Ag–Cu NPs	MB (5 nM)	1621	3 × 10^7^		23
Ag NPs	MB (1 mM to 1 pM)	1626	10^9^	7.21 × 10^−12^ M	This work
Ag NPs	MB (1 mM to 1 pM)	1394	10^9^		This work
Ag NPs	MB (1 mM to 1 pM)	1445	10^9^		This work
Ag nanodendrites	Ammonium nitrate (10^−6^ M)	1048	3.1 × 10^4^	∼350 nM	51
Ag–Cu NPs	Ammonium nitrate (5 mM)	1047	3.3 × 10^4^		23
Ag coated Si substrates decorated with AgAu bimetallic nanoparticles	Lead nitrate (10^−6^ M to 1 pM)	1047		10^−12^ M	47
Silver phosphate with bipyridine	Pb^2+^	1615	10^10^	0.09 × 10^−14^ M	52
Ag NPs	Lead nitrate (1 mM to 1 pM)	1045	10^10^	2.2 × 10^−12^ M	This work
Au NPs doped PMMA volcanic crater array	Cd^2+^ (10^−11^ M–10^−5^ M)	1586 (Cy3 reporter)	10^7^	10^−11^ M	49
Alizarin with Au NPs/3-mercaptopropionic acid (MPA)/2,6-pyridinedicarboxylic acid (PDCA)	Cd^2+^ (10–100 ppt)	1335 (Cd with Alizarin/Au NPs/MPA-PDCA)	10^7^		50
Ag NPs	Cadmium chloride (1 mM to 1 pM)	236	10^10^	12.1 × 10^−12^ M	This work

## 5 Conclusions

In summary, Ag NPs were successfully synthesized using ns PLAL, yielding surfactant-free, crystalline, and uniformly distributed plasmonic NPs. Structural and optical characterization confirmed the formation of spherical Ag nanoparticles with an average particle size of 21 ± 6 nm and a well-defined SPR peak at 407 nm, indicating strong plasmonic activity and controlled size distribution. High crystallinity of Ag NPs is confirmed by XRD and SAED. The absence of chemical stabilizers ensures minimal surface contamination, preserving the intrinsic plasmonic response of Ag facilitating direct analyte adsorption. SERS analysis of MB, Pb(NO_3_)_2_, and CdCl_2_ demonstrated the versatility and sensitivity of the laser-ablated Ag NPs substrates towards molecular and ionic analytes. High enhancement factor (∼10^9^ for pM MB and ∼10^10^ for Pb(NO_3_)_2_, and CdCl_2_) and low LOD, LOQ and RSD values discloses the ultra-sensitivity of Ag NPs as SERS substrates. Further, the stability of NPs can be improved by adding suitable stabilizers which will be future scope of the current work. The demonstrated ability to detect organic dyes, nitrate ions, and heavy metal salts with high sensitivity using unfunctionalized Ag NPs as SERS substrates highlights the potential of laser-ablated Ag NPs for multifunctional sensing platforms. Such substrates are particularly attractive for environmental and chemical sensing applications due to their simplicity, reproducibility, and high sensitivity without the need for chemical functionalization.

## Author contributions

Pavithra K – writing the original draft, methodology, investigation; S. Swaroop – visualization, review, editing, resources; Venugopal Rao Soma – review, editing, conceptualisation; Anusha P. T – review, editing, validation, supervision, resources, project administration, conceptualisation.

## Conflicts of interest

There are no conflicts to declare.

## Supplementary Material

RA-016-D6RA05127H-s001

## Data Availability

The data that support the findings of this study are available upon request. Supplementary information (SI): the absorption spectra of analytes, SERS signal homogeneity graphs at different concentrations, SERS mapping images at 1 pM concentration, AEF and intensity values at all concentrations and stability studies. See DOI: https://doi.org/10.1039/d6ra05127h.
