# Supplementary material for: SERS-based sensing of heavy metal ions and methylene blue using nanosecond laser ablated silver nanoparticles
Source: RSC Adv. 2026 Aug 25;16(42):50306–17. doi: 10.1039/d6ra05127h (PMC13504420; doi:10.1039/d6ra05127h)
Supplement: Supplementary file 1 [file RA-016-D6RA05127H-s001.pdf]

Supplementary Information (SI) for RSC Advances.
This journal is © The Royal Society of Chemistry 2026

Supporting Information

SERS-based Sensing of Heavy Metal Ions and Methylene Blue Using Nanosecond Laser Ablated Silver Nanoparticles

Pavithra K[1], S. Swaroop[1*], Venugopal Rao Soma[3] and Anusha P T[1,2*]

[1]Department of Physics, School of Advanced Sciences, Vellore Institute of Technology (VIT), Vellore 632014, Tamil Nadu, India

[2]Department of Physics, Kannur University, Swami Anandatheertha Campus-Payyanur, Kannur 670327, India

[3]School of Physics and DIA-CoE (Formerly ACRHEM), University of Hyderabad, Hyderabad 500046, India

***Corresponding Authors: anushapt@kannuruniv.ac.in, sathyaswaroop.n.r@vit.ac.in**

Table of Contents

S. No.	Contents	Page Numbers
**1**	UV-Visible absorption data of MB, lead nitrate and cadmium chloride	S3
**2**	SERS signal homogeneity graphs at different concentrations of MB, lead nitrate and cadmium chloride. SERS mapping data of (a)Ag-MB (b)Ag-$Pb(NO_3)_2$ (c)Ag-$CdCl_2$	S4-S5
**3**	Tables with analytical enhancement factor (AEF) calculations of MB, lead nitrate and cadmium chloride	S6-S7

**4**	Stability studies: UV-visible absorption and the SERS performance	S7-S8

1. UV-Visible absorption spectroscopic studies

1.1. Methylene Blue (MB)

Fig. S1. shows the absorption spectrum of MB dispersed in distilled water at 1.5 mM concentration. The absorption peaks in ultraviolet region at 245 nm and 292 nm are associated with substituted benzene rings. The most intense peak at 663 nm is due to MB monomer with shoulder peak at 612 nm is due to MB dimer.[1,2]

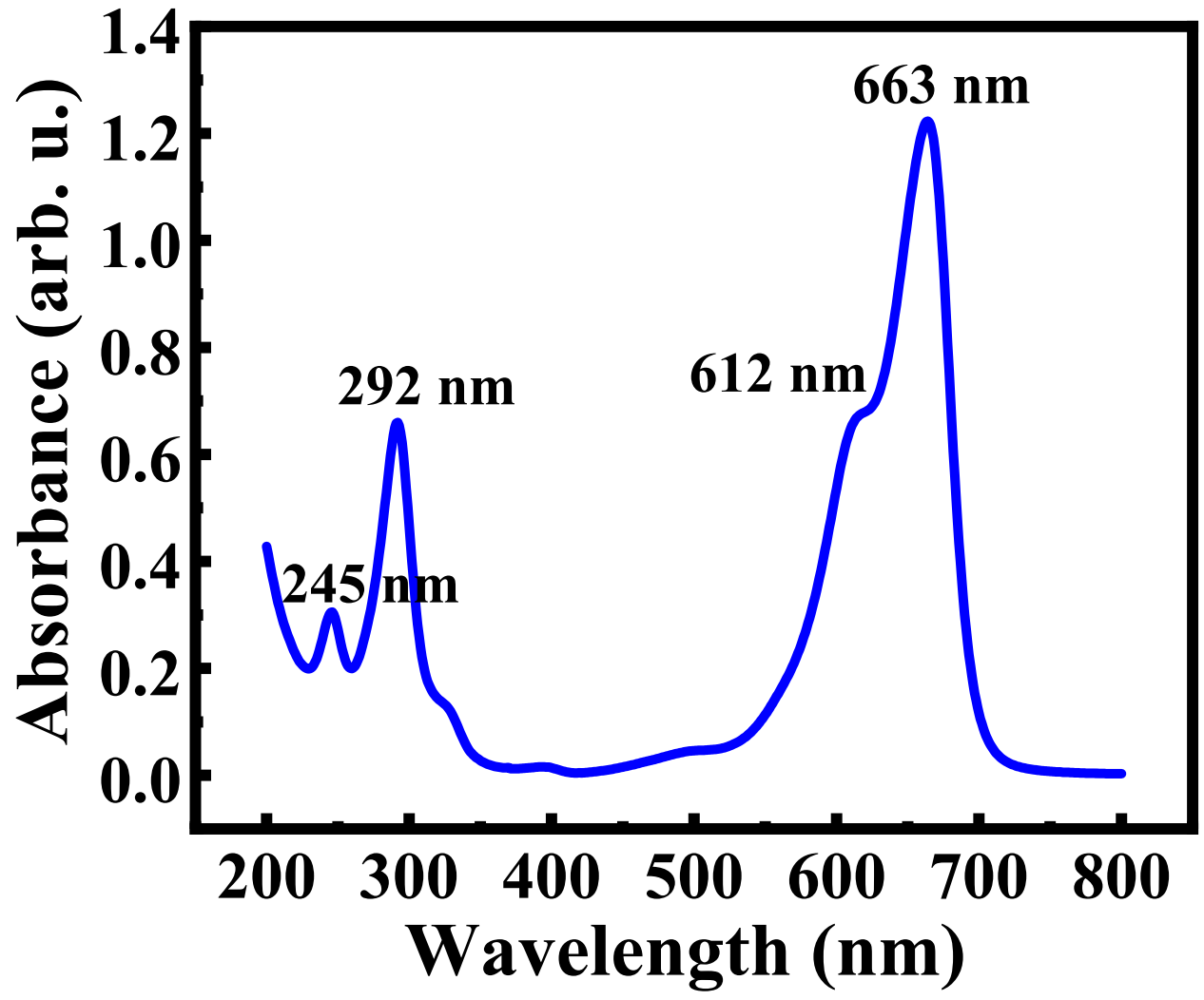

Fig. S1. UV-visible absorption spectrum of methylene blue.

1.2. Lead nitrate and cadmium chloride

Absorption spectra of lead nitrate and cadmium chloride dispersed in distilled water at 0.02 M concentration is represented in fig. S2. For lead nitrate, the peak at 241 nm corresponds to π-π* transition mixed with ligand-to-metal charge transfer (LMCT) involving nitrate oxygen atoms and Pb^{2+} ions. Absorption peak at 299 nm is due to n-π* transition of nitrate ion. The maximum absorbance of cadmium chloride at 210 nm due to LMCT (an electron from chloride ligand orbital is excited to an empty orbital of the Cd^{2+}). Because of filled d-orbitals of Cd^{2+} ([Kr]$4d^{10}$) d-d transitions are not possible for cadmium chloride.[3,4]

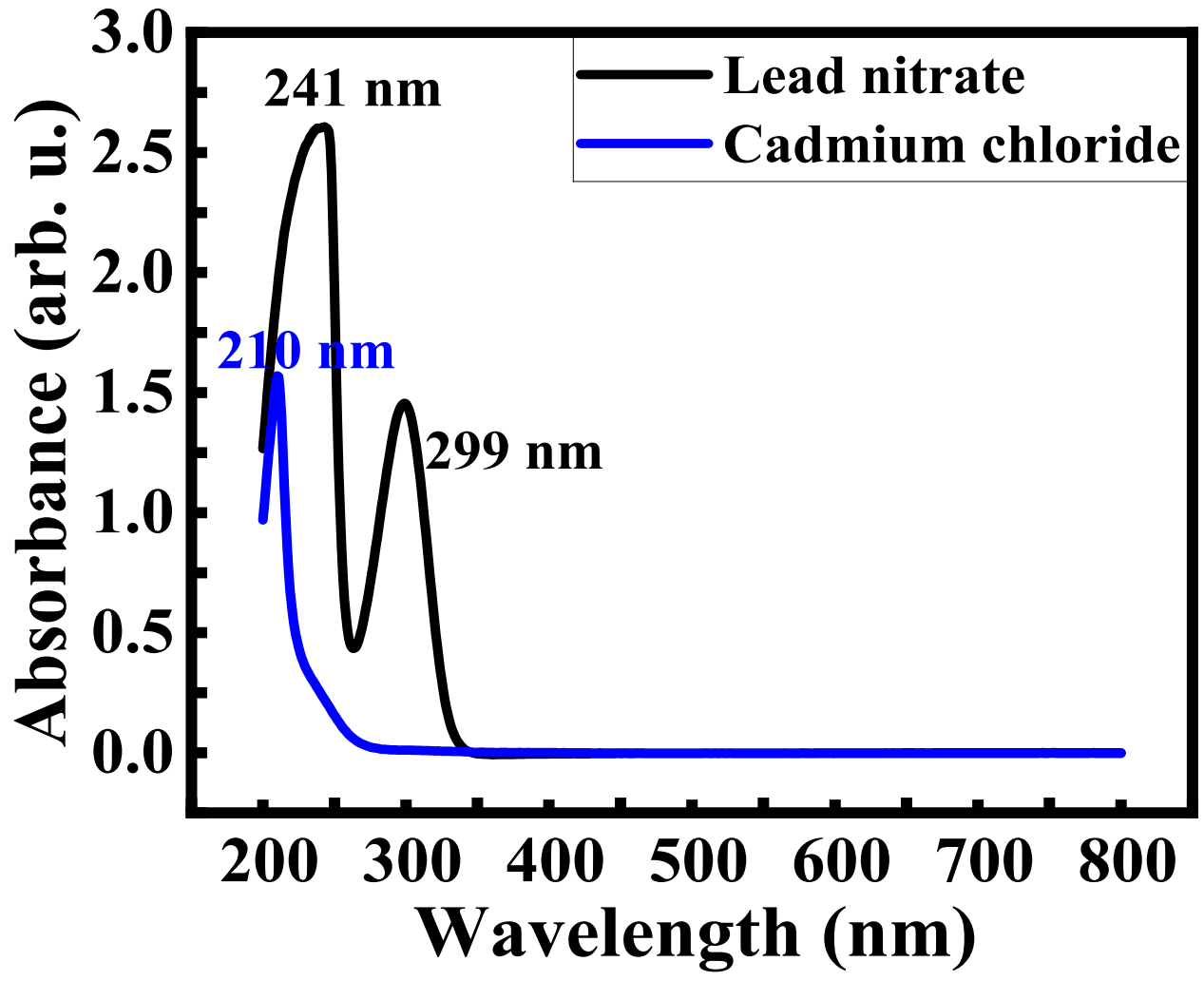

Fig. S2. UV-visible absorption spectra of lead nitrate and cadmium chloride

2. SERS signal homogeneity graphs at different concentrations

SERS spectra were taken at five different spots of the substrate to check the homogeneity of the signal throughout the surface. The spectra of five different concentrations of each of analyte MB, lead nitrate and cadmium chloride taken at five random points is represented in figures S3, S4 and S5 respectively. Figure S6 represents the SERS mapping images of methylene blue, $Pb(NO_3)_2$ and $CdCl_2$ at 1 pM concentration with Ag NPs, which confirms the homogeneity of the substrates even at 1 pM concentration. The figures show almost same signal quality which emphasis that the substrate is uniform.

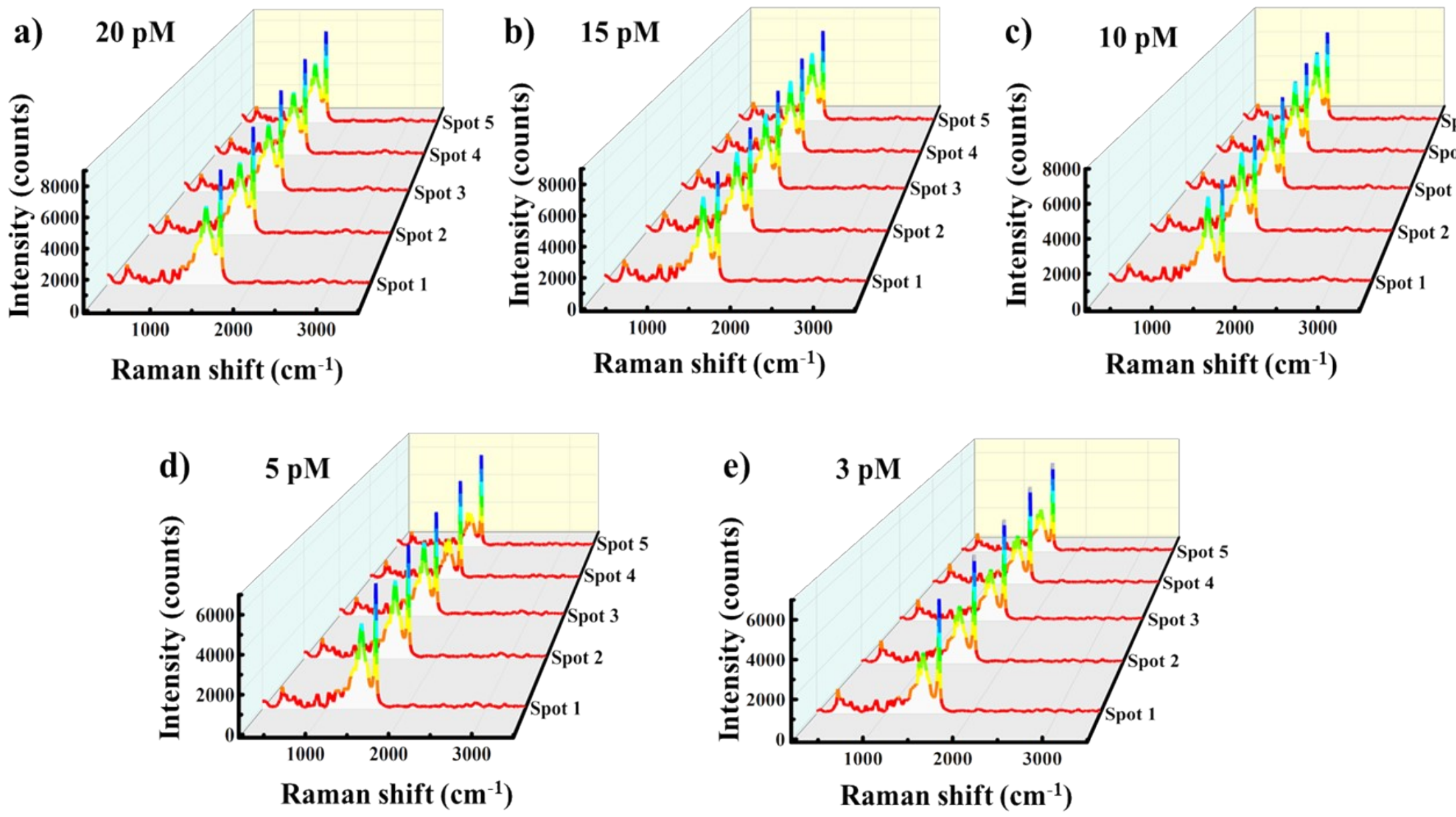

Fig. S3. SERS signal homogeneity of methylene blue (MB) a) 20 pM, b) 15 pM, c) 10 pM, d) 5 pM and e) 3 pM at 5 random spots

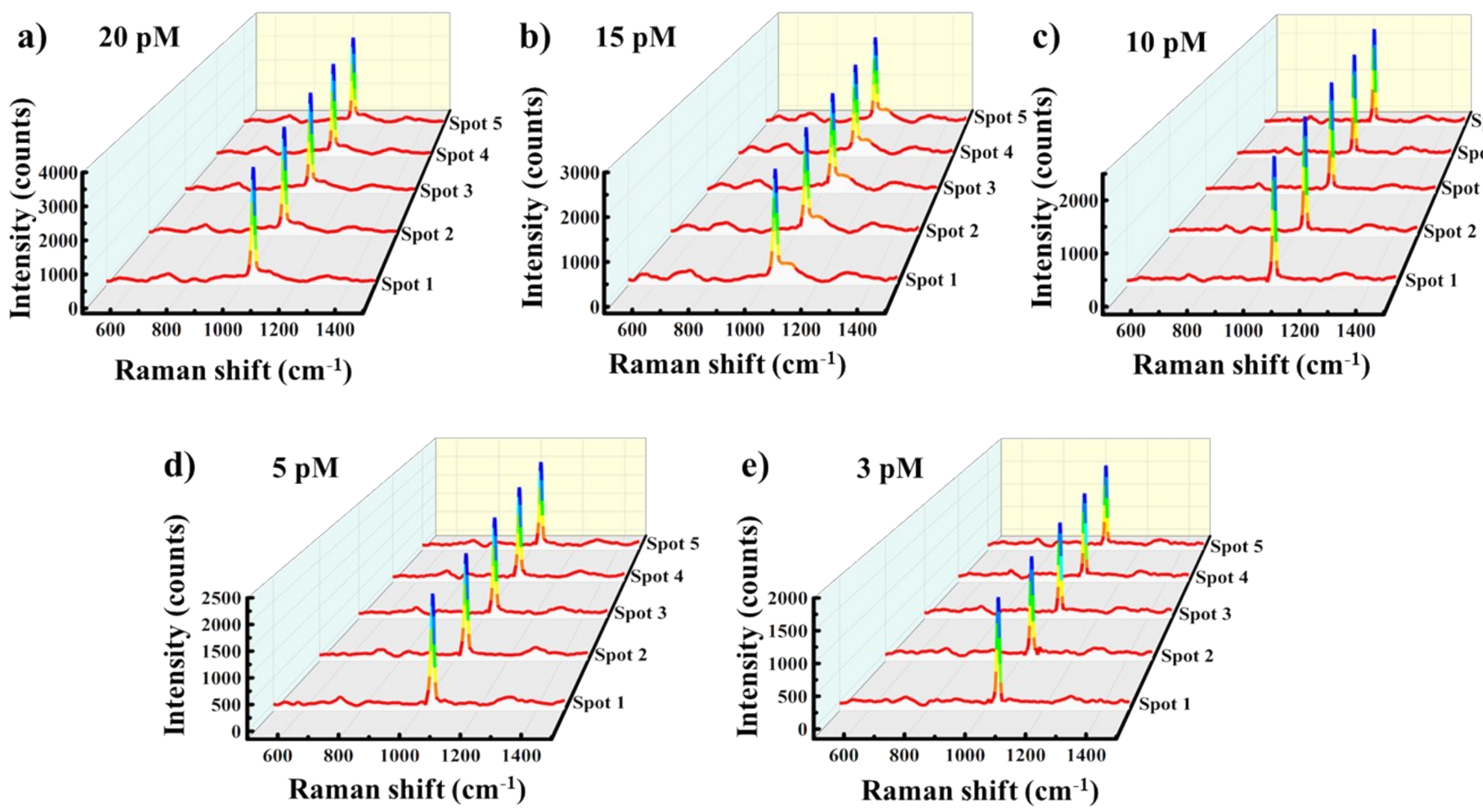

Fig. S4. SERS signal homogeneity of lead nitrate a) 20 pM, b) 15 pM, c) 10 pM, d) 5 pM and e) 3 pM at 5 random spots.

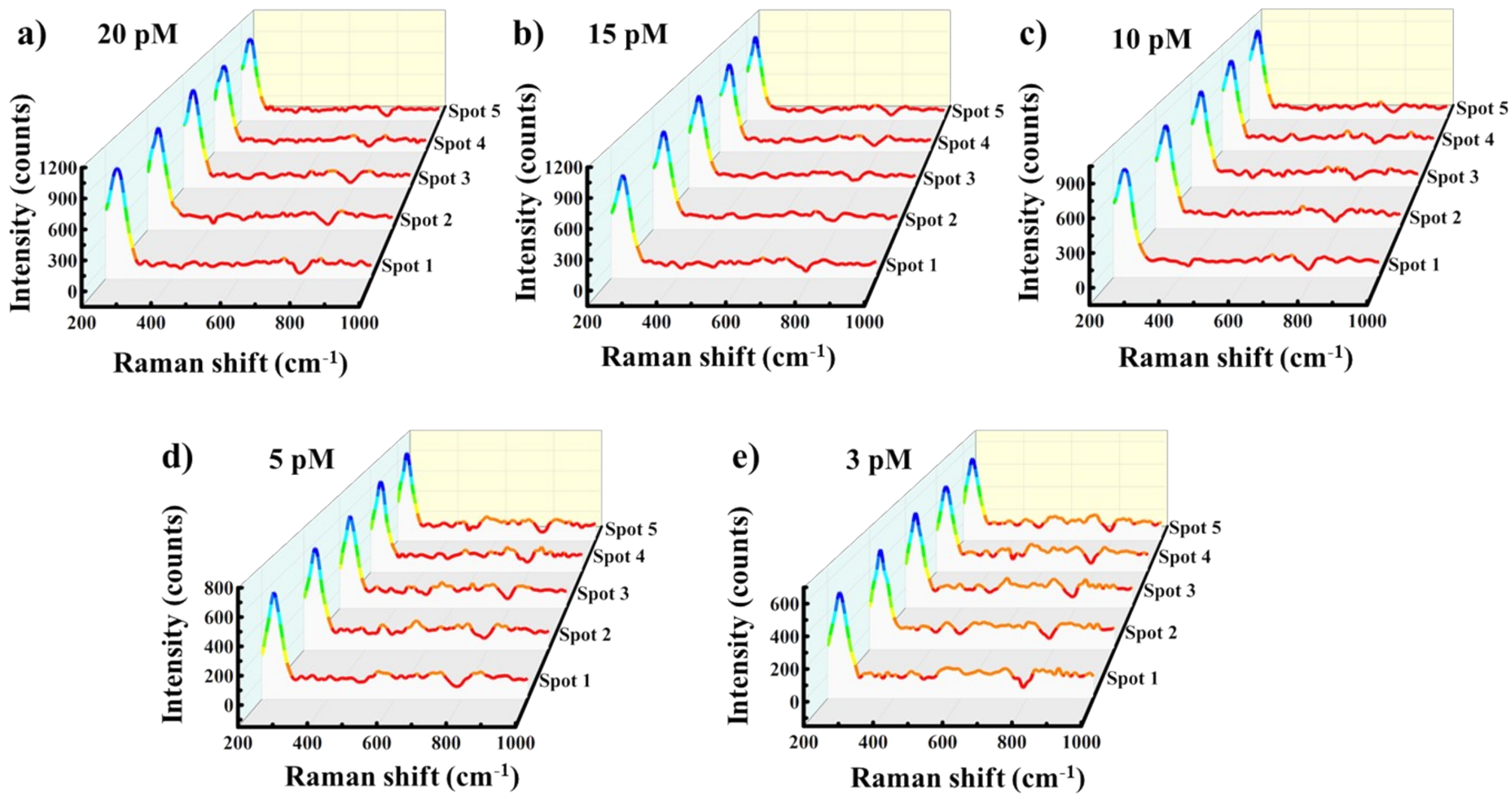

Fig. S5. SERS signal homogeneity of cadmium chloride a) 20 pM, b) 15 pM, c) 10 pM, d) 5 pM and e) 3 pM at 5 random spots

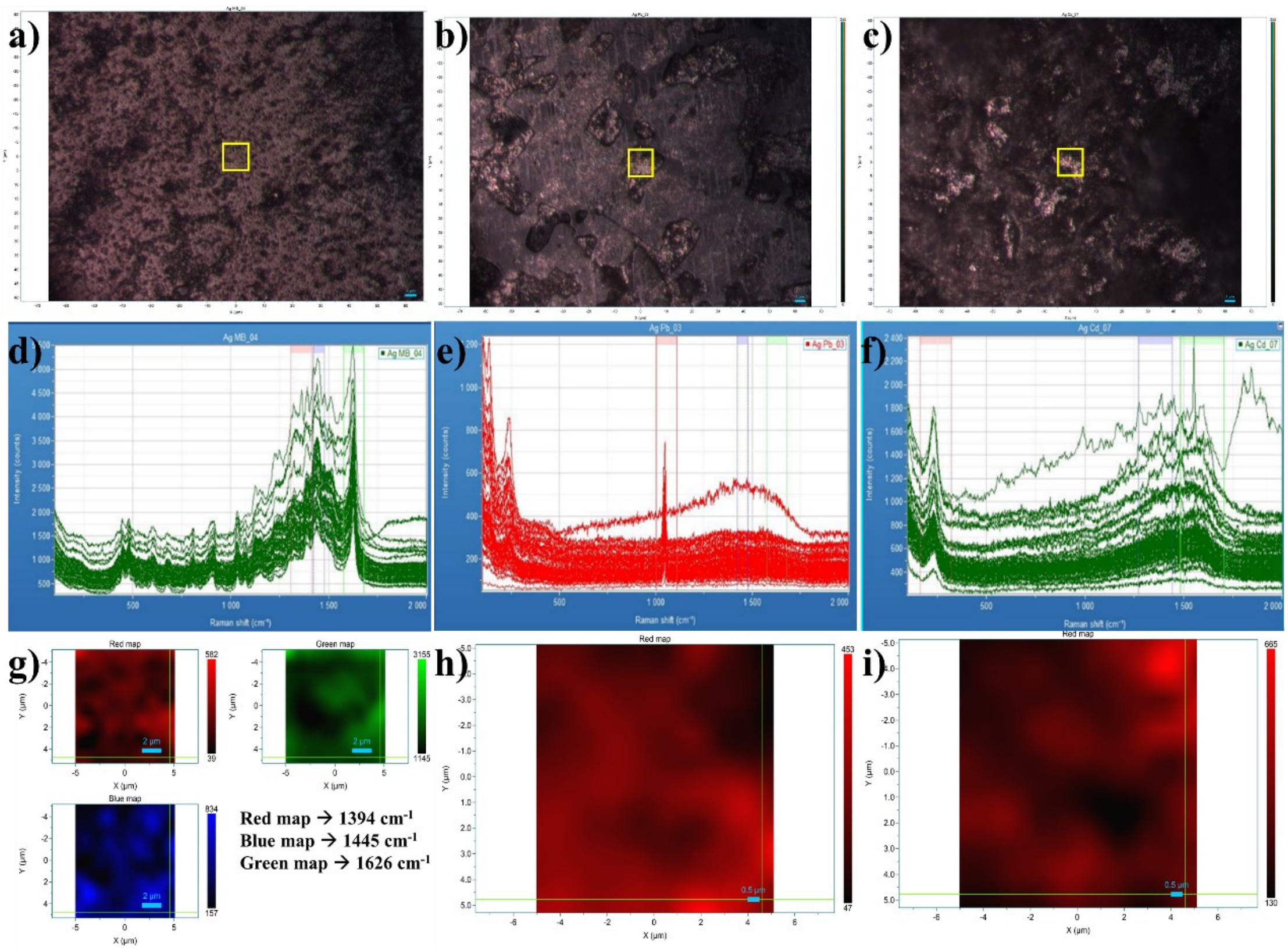

Fig. S6. Surface image of mapped area of (a)Ag-MB (b)Ag-$Pb(NO_3)_2$ (c)Ag-$CdCl_2$; SERS spectra of (d)Ag-MB (e) Ag-$Pb(NO_3)_2$ (f) Ag-$CdCl_2$; SERS intensity mapping of (g)Ag-MB at 1394 cm^{-1},1445 cm^{-1}, 1626 cm^{-1} (h) Ag-$Pb(NO_3)_2$ peak at 1045 cm^{-1} (i) Ag- $CdCl_2$ peak at 236 cm^{-1}. The analyte concentrations were taken at 1 pM.

3. Analytical Enhancement Factor (AEF)

Table S1. AEFs of MB at different concentrations with Ag NPs

**Concentration of MB**	I_{SERS}	**AEF**	I_{SERS}	**AEF**	I_{SERS}	**AEF**
	**1626 cm^{-1}**		**1394 cm^{-1}**		**1445 cm^{-1}**	
1 mM	9015	3.4	3420	4.17	2842	5.6
1 μM	8746	3.3×10^3	3348	4.08×10^3	2570	5.09×10^3
15 nM	8606	0.2×10^6	3654	0.29×10^6	3542	0.46×10^6
1 nM	8239	3.1×10^6	4093	4.99×10^6	6877	13.62×10^6
20 pM	7734	0.14×10^9	3792	0.23×10^9	5148	0.51×10^9
15 pM	7595	0.19×10^9	3826	0.31×10^9	5854	0.77×10^9
10 pM	6446	0.24×10^9	3039	0.37×10^9	5197	1.02×10^9
5 pM	6193	0.47×10^9	2631	0.64×10^9	3517	1.39×10^9
3 pM	6069	0.77×10^9	2585	1.05×10^9	3009	1.98×10^9
1 pM	5333	2.04×10^9	1864	2.27×10^9	1864	3.69×10^9

Table S2. AEFs of $Pb(NO_3)_2$ at different concentrations with Ag NPs

**Concentration of lead nitrate**	I_{SERS}	**AEF**
	1045 cm^{-1}	
1 mM	8103	0.03
1 μM	5574	2.08×10^4
15 nM	5308	0.132×10^7
1 nM	3487	1.303×10^7
20 pM	3387	0.063×10^{10}
15 pM	2531	0.063×10^{10}
10 pM	2296	0.085×10^{10}
5 pM	2129	0.159×10^{10}
3 pM	1532	0.19×10^{10}
1 pM	1269	0.474×10^{10}

Table S3. AEFs of $CdCl_2$ with Ag NPs at different concentrations

Concentration of Cadmium chloride	I_{SERS}	AEF
	236 cm^{-1}	
1 mM	2775	0.074
1 µM	1475	3.97×10^4
15 nM	1313	0.235×10^7
1 nM	1072	2.885×10^7
20 pM	975	0.131×10^{10}
15 pM	955	0.171×10^{10}
10 pM	878	0.236×10^{10}
5 pM	655	0.352×10^{10}
3 pM	546	0.489×10^{10}
1 pM	481	1.294×10^{10}

4. Stability studies

4.1 UV-Visible absorption spectroscopy of silver nanoparticles (Ag NPs)

Fig. S7 shows the absorption spectra of Ag nanoparticles which were recorded right after the synthesis and after two months. The SPR peak of synthesised Ag NPs at 407 nm is shifted to 406 nm with a slight decrease in absorbance intensity after two months of storage.

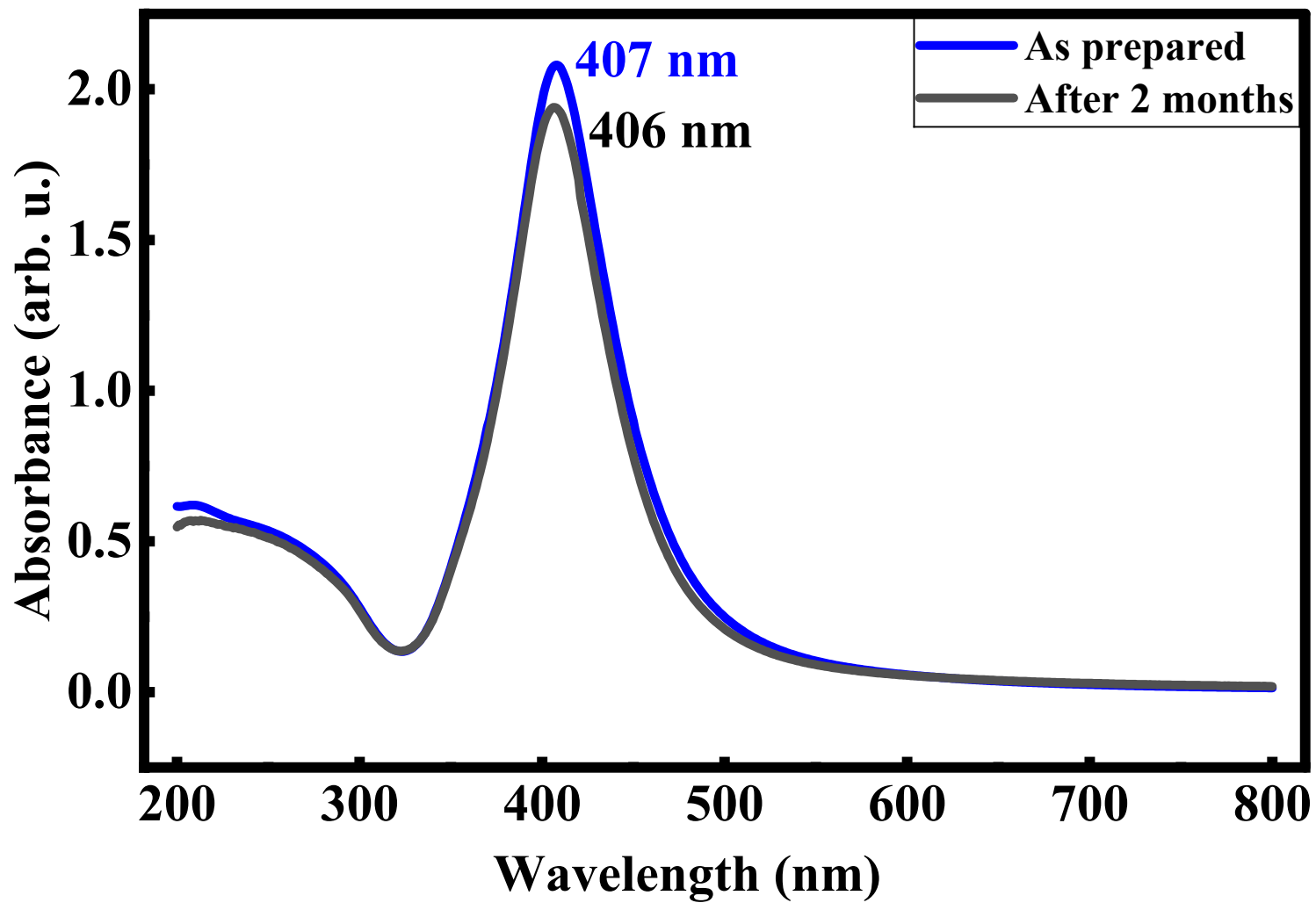

Fig. S7. UV-visible absorption spectra of Ag nanoparticles as prepared (blue line) and after two months (black line)

4.2 SERS studies

Fig. S8. shows the SERS spectra MB, $Pb(NO_3)_2$ and $CdCl_2$ at a concentration of 1 pM as prepared and after 2 months of storage using Ag NPs substrates. The changes in SERS intensities and the peak shift of the SERS substrates were observed after storage of two months. Table S4. shows the AEF values at concentration of 1 pM of the SERS substrates as prepared and after storage of two months.

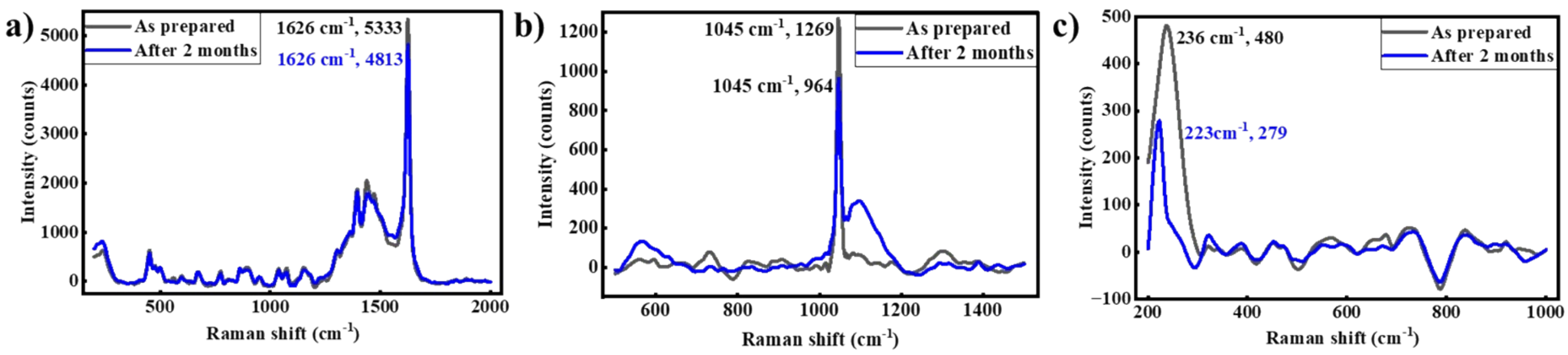

Fig. S8. SERS spectra of (a) MB, (b) $Pb(NO_3)_2$ and (c) $CdCl_2$ at a concentration of 1 pM as prepared (black line) and after 2 months (blue line) using Ag NPs substrates, respectively.

Table S4. AEFs of MB, $Pb(NO_3)_2$ and $CdCl_2$ at a concentration of 1 pM as prepared and after 2 months of storage using Ag NPs substrates

Analyte (1 pM concentration)	Analytical Enhancement Factor (AEF)		
	MB (1626 cm^{-1})	Lead nitrate (1045 cm^{-1})	Cadmium chloride (236 cm^{-1})
As prepared	2.04×10^9	0.47×10^{10}	1.29×10^{10}
After 2 months	1.84×10^9	0.36×10^{10}	0.75×10^{10}
Percentage reduction in AEF	10%	24%	42%

References

1 . S. Mondal, Ma. E. De Anda Reyes and U. Pal, *RSC Adv.*, 2017, *7*, 8633–8645.
2 . I. Khan, K. Saeed, I. Zekker, B. Zhang, A. H. Hendi, A. Ahmad, S. Ahmad, N. Zada, H. Ahmad, L. A. Shah, T. Shah and I. Khan, *Water*, 2022, **14**, 242.
3 . G. L. Miessler, P. J. Fischer and D. A. Tarr, *Inorganic Chemistry*, Pearson, Boston, 5th edn, 2014.
4 . D. A. Skoog, F. J. Holler and S. R. Crouch, *Principles of Instrumental Analysis*, Cengage Learning, Boston, 7th edn.
